# The Native Orthobunyavirus Ribonucleoprotein Possesses a Helical Architecture

**DOI:** 10.1128/mbio.01405-22

**Published:** 2022-06-28

**Authors:** Francis R. Hopkins, Beatriz Álvarez-Rodríguez, George R. Heath, Kyriakoulla Panayi, Samantha Hover, Thomas A. Edwards, John N. Barr, Juan Fontana

**Affiliations:** a School of Molecular and Cellular Biology, Faculty of Biological Sciences, University of Leeds, Leeds, United Kingdom; b Astbury Centre for Structural Molecular Biology, University of Leeds, Leeds, United Kingdom; c School of Physics and Astronomy, Faculty of Engineering and Physical Sciences, University of Leeds, Leeds, United Kingdom; MRC-University of Glasgow Centre for Virus Research; University of Pennsylvania

**Keywords:** orthobunyavirus, ribonucleoprotein, structure, bunyavirus

## Abstract

The *Bunyavirales* order is the largest group of negative-sense RNA viruses, containing many lethal human pathogens for which approved anti-infective measures are not available. The bunyavirus genome consists of multiple negative-sense RNA segments enwrapped by the virus-encoded nucleocapsid protein (NP), which together with the viral polymerase form ribonucleoproteins (RNPs). RNPs represent substrates for RNA synthesis and virion assembly, which require inherent flexibility, consistent with the appearance of RNPs spilled from virions. These observations have resulted in conflicting models describing the overall RNP architecture. Here, we purified RNPs from Bunyamwera virus (BUNV), the prototypical orthobunyavirus. The lengths of purified RNPs imaged by negative staining resulted in 3 populations of RNPs, suggesting that RNPs possess a consistent method of condensation. Employing microscopy approaches, we conclusively show that the NP portion of BUNV RNPs is helical. Furthermore, we present a pseudo-atomic model for this portion based on a cryo-electron microscopy average at 13 Å resolution, which allowed us to fit the BUNV NP crystal structure by molecular dynamics. This model was confirmed by NP mutagenesis using a mini-genome system. The model shows that adjacent NP monomers in the RNP chain interact laterally through flexible N- and C-terminal arms only, with no longitudinal helix-stabilizing interactions, thus providing a potential model for the molecular basis for RNP flexibility. Excessive RNase treatment disrupts native RNPs, suggesting that RNA was key in maintaining the RNP structure. Overall, this work will inform studies on bunyaviral RNP assembly, packaging, and RNA replication, and aid in future antiviral strategies.

## INTRODUCTION

The *Bunyavirales* order of segmented, negative-sense RNA viruses contains over 500 named isolates divided into 12 families (1), five of which include human and animal pathogens. One of these families, *Peribunyaviridae*, comprises arboviruses and is further divided into four genera, with the *Orthobunyavirus* genus being the largest. Important animal-infecting orthobunyaviruses include Schmallenberg virus (SBV) and Akabane virus, which both cause stillbirths and deformities in ruminants (2, 3). Important human-infecting orthobunyaviruses include Oropouche, Jamestown Canyon, and La Crosse (LACV) viruses, which are associated with acute febrile illnesses with frequent central nervous system infiltration; and Ngari virus, which is responsible for fatal hemorrhagic fever (4–6). Recent reports of the newly identified Cristoli orthobunyavirus and the re-emergence of Umbre orthobunyavirus as agents of fatal encephalitis, confirm the risk posed by orthobunyaviruses to human health (7, 8). While vaccines have been developed against several animal-infecting orthobunyaviruses, including SBV (2, 9), no FDA-approved vaccines or therapies are available for those infecting humans. As insect habitats shift in response to climate change, arboviruses pose a growing threat and present a pressing need for new strategies in vaccine and antiviral design (10).

Bunyamwera virus (BUNV) is the prototypic orthobunyavirus that serves as a tractable model for the *Bunyavirales* order. BUNV virions are pleiomorphic and include a host-derived lipid envelope coated in virus-encoded glycoprotein spikes (11), surrounding the RNA genome comprising small (S), medium (M), and large (L) segments. Each segment is encapsidated by multiple copies of the nucleocapsid protein (NP) to form a ribonucleoprotein (RNP), which associates with the virus-encoded RNA-dependent RNA polymerase (RdRp; L). RNPs serve as the active templates for all viral RNA synthesis activities and represent the form in which the RNA segments are packaged into virions.

The BUNV NP comprises a globular core with N- and C-terminal lobes bisected by a positively charged RNA-binding groove (12–17), accommodating approximately 11 RNA bases. The N- and C-termini protrude from the core to form ‘arms’ that mediate homotypic oligomerization, and this overall arrangement is shared by NPs from SBV, LACV, and Leanyer orthobunyaviruses (12–17). Recombinantly expressed orthobunyavirus NP crystallizes as closed, tetrameric, or hexameric rings (17) and this oligomeric promiscuity was confirmed by mass spectrometry and electron microscopy (EM), which revealed oligomers of increasing size from trimer to octamer (15, 18, 19).

**FIG 1:**
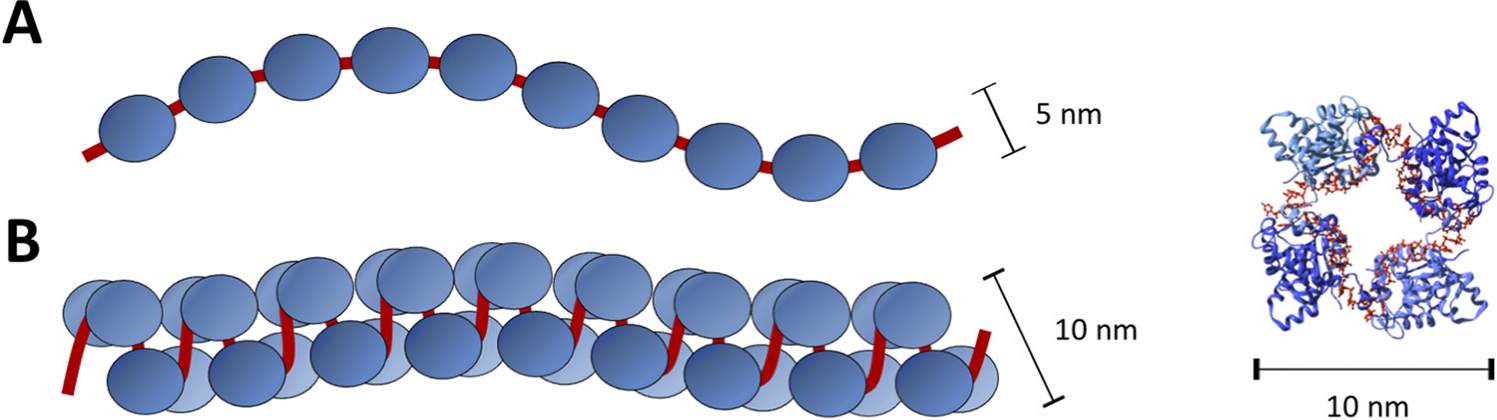
Proposed models of NP organization within orthobunyavirus RNP. (A) Monomer-based ‘beads on a string’ and (B) tetramer-based helix alongside the crystal structure of tetrameric BUNV NP.

Previous EM analyses of native RNPs released from virions showed they were highly flexible and resulted in conflicting models that described their overall architecture (Fig. 1). One model proposed that RNPs adopted a ‘beads on a string’ organization, based on EM observations of flexible RNPs with a diameter of single NP monomers (14, 15). The alternative model proposed that RNPs adopted a relaxed helical architecture, which was supported by EM observations of RNPs that exhibited a staggered positioning of monomers with a cross-sectional width corresponding to two NPs and interpreted as a helix of ~4 NPs per turn (12, 13), and by the crystallization of RNA-free LACV NP oligomers in a helical array (13).

EM observation of native RNPs from the *Arenaviridae*, *Phenuiviridae*, and *Nairoviridae* families within the *Bunyavirales* order also revealed a flexible architecture (20–22). In contrast, a high-resolution cryo-electron microscopy (cryo-EM) model of a rod-like RNP from a member of the *Hantaviridae* family reconstituted from recombinant protein, revealed a compact and rigid helical architecture, maintained by both lateral and longitudinal NP-NP interactions through the helix (23). However, to date, limited structural information from native RNPs exists for any member of the *Bunyavirales* order.

Here, we purified native RNPs from infectious virions and characterized them by microscopy approaches. The gross characteristics of RNPs were determined by negative staining and subsequently, cryo-electron tomography (cryo-ET) was performed. Particles within the cryo-ET data were picked and subject to subtomogram averaging (STA) to produce a three-dimensional model of the NP portion of the RNP filament, conclusively showing that BUNV RNPs are helical. Based on a cryo-EM average at 13 Å resolution and subsequent fitting of NP crystal structures by molecular dynamics, we were able to generate a pseudo-atomic model of the NP portion of the RNP. Our model suggests that the helical arrangement of BUNV RNP is dictated by lateral NP-NP interactions and does not involve longitudinal NP-NP interactions, providing a molecular explanation of the RNP flexibility. Furthermore, we showed that the RNA was key in maintaining the structure of the RNPs. This work will inform future studies into the mechanisms of viral processes, such as RNP assembly and packaging.

## RESULTS

### BUNV RNP purification.

**FIG 2:**
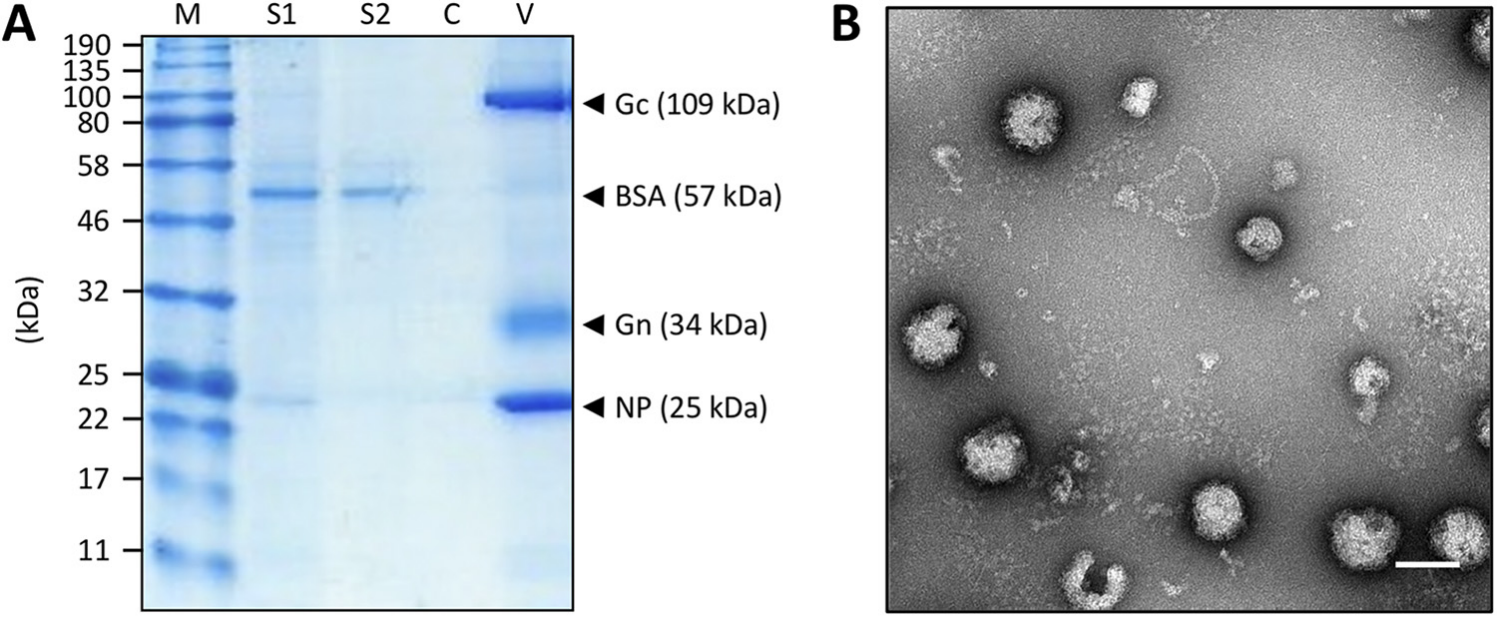
Purification of BUNV. (A) SDS-PAGE analysis showed an increased concentration of virus after pelleting through a sucrose cushion, with high purity and removal of bovine serum albumin (BSA) contaminants. M = marker, S1 = supernatant prepurification, S2 = clarified supernatant prepurification, C = cushion post purification, and V = resuspended viral pellet. (B) Negative staining EM confirmed purity and abundance of virions. Scale bar: 100 nm.

**FIG 3:**
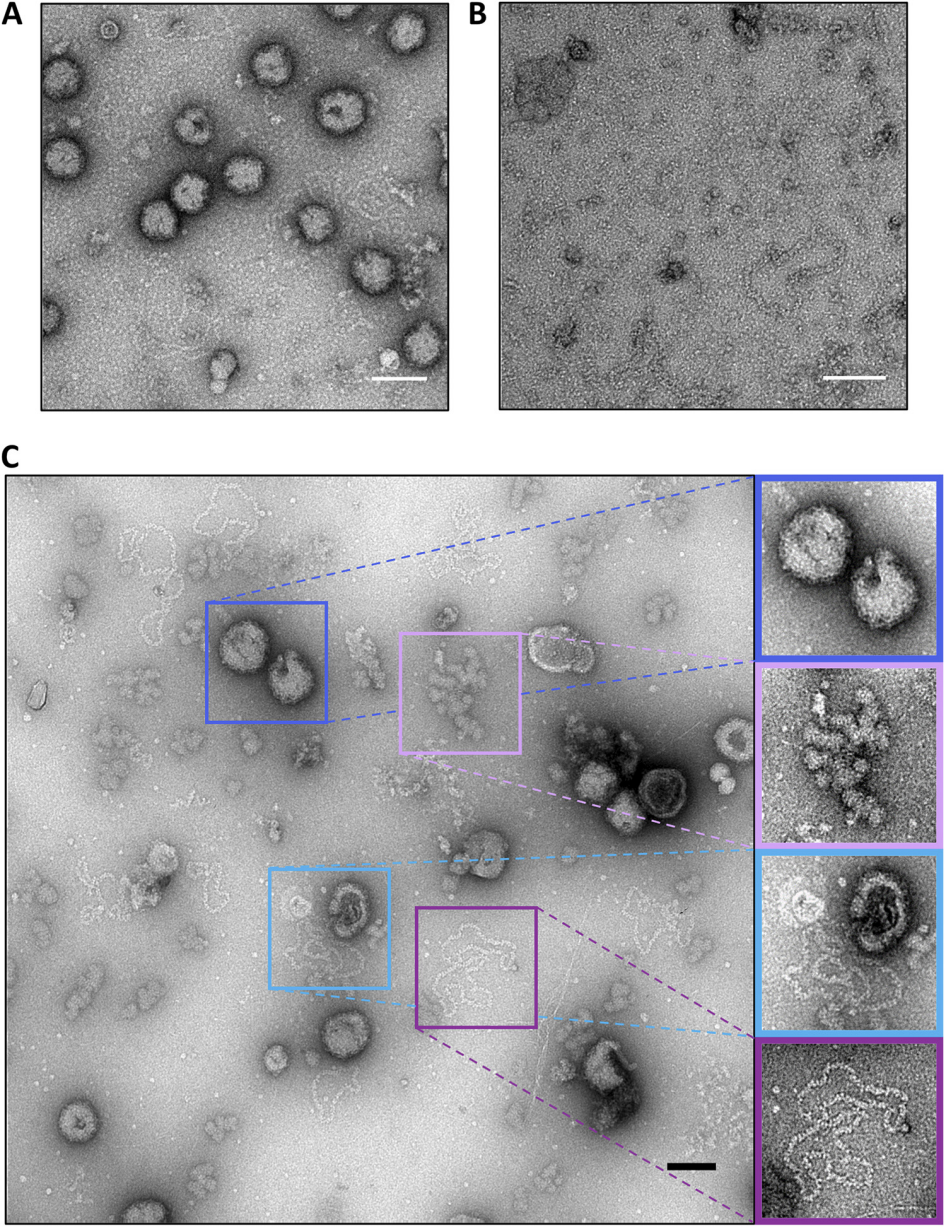
Methods of BUNV RNP release, examined by negative staining. (A) Sucrose washes were not reproducible and often failed to lyse virions. (B) Washing with detergent was effective at disrupting virions but produced a high level of background signal. (C) Freeze/thaw was highly effective for the release of viral RNPs. From top to bottom, insets show mostly intact viruses, debris attributed to disrupted viral membranes, RNPs spilling from their parent virus, and discrete RNPs released from virions. Scale bars, 100 nm.

**FIG 4:**
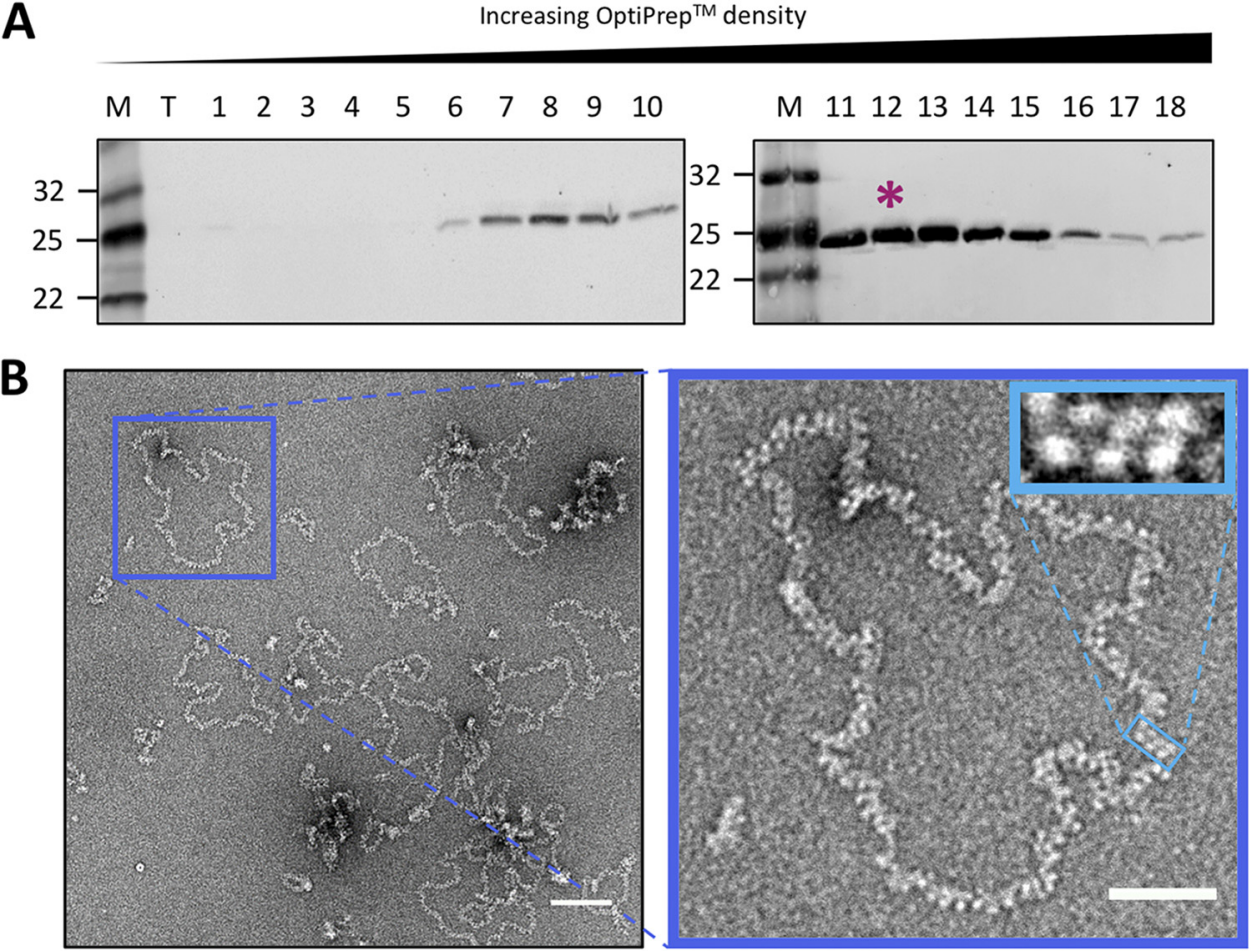
BUNV RNP purification and negative staining EM. (A) Fractionation and western blotting showed the location of RNPs within a continuous density gradient (asterisk). (B) Negative staining EM confirmed the abundance and purity of RNP filaments, and regions of the apparent helical organization were apparent (inset). Scale bar, 100 nm in the main and 50 nm in the inset.

**FIG 5:**
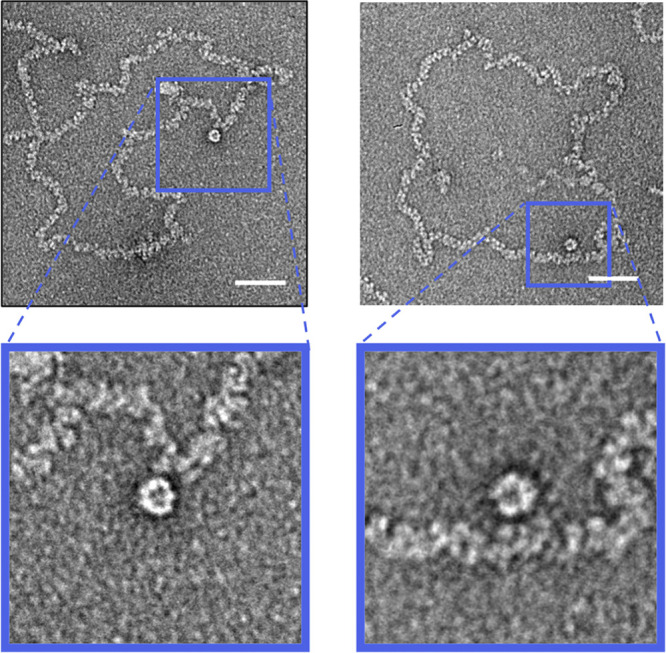
Putative polymerase molecules within BUNV RNP preparations. During analysis of negatively stained BUNV RNPs, a population of large structures with resemblance to viral polymerases was found, often in association with RNP filaments. Scale bars, 50 nm.

To structurally characterize native BUNV RNPs, virion purification and RNP isolation were optimized. BUNV was purified from infected cell supernatants by ultracentrifugation, with purity assessed by SDS-PAGE (Fig. 2A) and negative staining EM (Fig. 2B), which revealed intact as well as disrupted virions from which viral RNPs were released. RNPs were purified from freeze-thawed virions by ultracentrifugation (adapted from (12)) after testing different methods to release RNPs (Fig. 3), with RNP-containing fractions detected by western blotting (Fig. 4A). Subsequent analysis by negative staining EM revealed abundant, filamentous RNPs (Fig. 4B) with a uniform width along their entire lengths that corresponded to two NP monomers. These monomers adopted a staggered arrangement consistent with a helical conformation (Fig. 4B inset), and there was no indication of a ‘beads on a string’ morphology. Any disruption to the helical conformation was only apparent at the sharpest (≥90^°^) bends in the filament. Of note, almost all observed RNPs were circularized, and linear filaments were rare (less than one percent of the imaged RNPs). Although the RNA itself might not be resolved by negative staining, we expected to visualize a region along with the RNP where the NP organization was different, distinguishing the segment ends from nonterminal regions. However, no such region common to all segments was identified, potentially due to the limited resolution of negative staining EM. Interestingly, some RNPs contained an associated density potentially compatible with that of the viral RdRp (Fig. 5). These densities lack the additional globular domains appended to the ring-like structures reported for RdRps from negative-sense RNA viruses (24, 25), but they do resemble recent images of reconstituted polymerase-associated RNPs from an arenavirus (26). The fact that most RNPs lacked density for a putative RdRp suggested they became dissociated during the purification process. We do not know at what point of the purification process the RdRps were lost, although one possibility is that the low salt concentration used in our buffers may have caused some degree of polymerase dissociation. The RdRp might also be lost during grid preparation due to interactions with the air-water interface.

**FIG 6:**
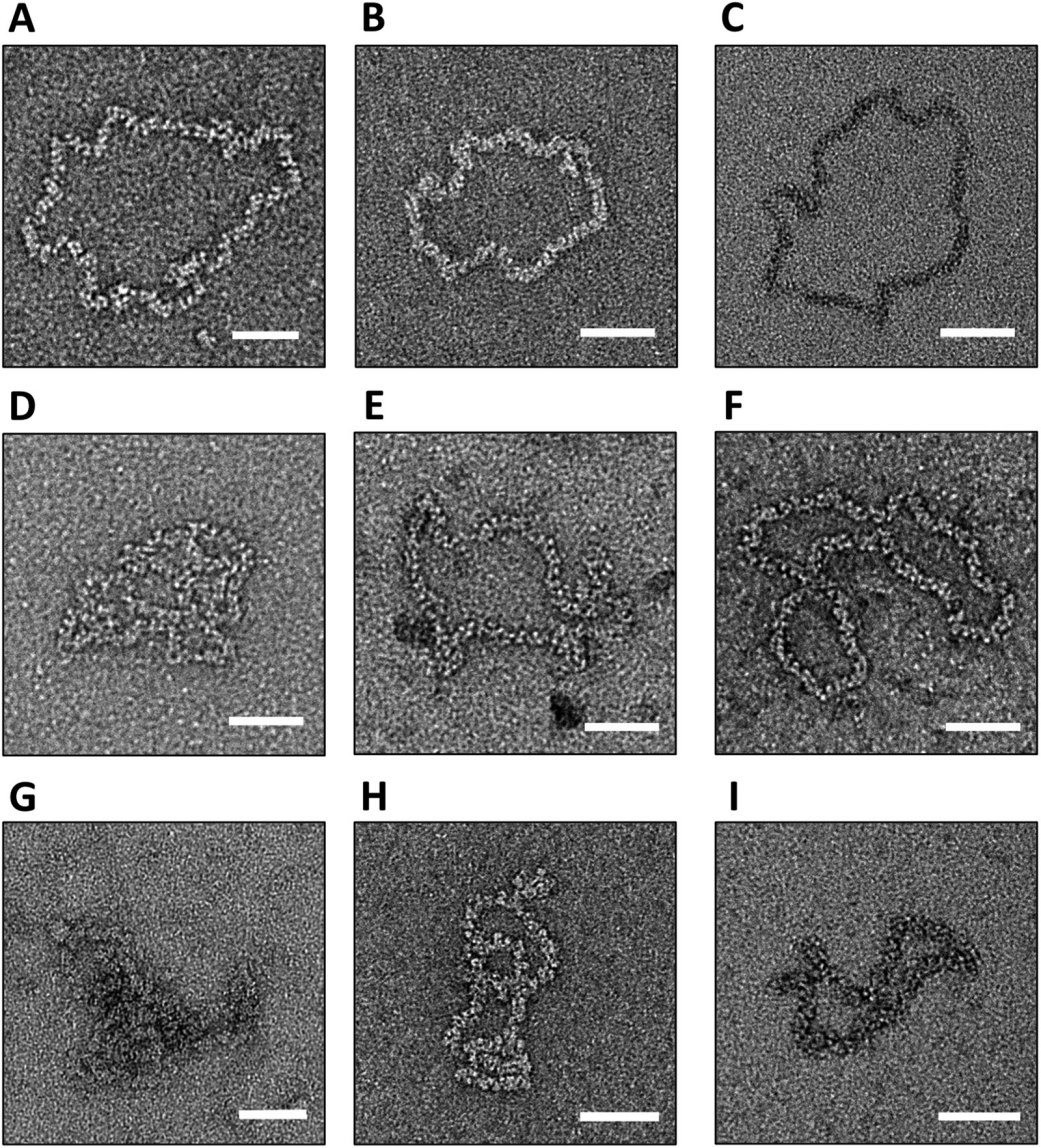
Visualization of purified BUNV RNPs after buffer exchange into different conditions. (A) TNE with no salt. (B) TNE with 25 mM NaCl. (C) TNE with 50 mM NaCl. (D) TNE with the addition of MgCl_2_. (E) TNE with the addition of DTT. (F) TNE with KCl substituted for NaCl. (G) TNE with the addition of antisera. (H) TNE pH 6.4. (I) TNE pH 8.0. Scale bars: 50 nm.

To aid in the alignment and averaging of filaments, different buffers were explored for their effect on RNP straightness. Despite the effects being small, RNPs appeared moderately straighter in lower salt concentrations than the 200 mM in the original TNE buffer. Thus, a reduced-salt form of the TNE buffer containing 25 mM NaCl was selected for subsequent EM analysis. Across all buffers the RNP filaments appeared to maintain a uniform width, suggesting that there was no difference in the overall helical organization (Fig. 6). We could not exclude the possibility that the purification process might have influenced the RNP structure. However, we note that similar purification methods have been employed to purify influenza virus RNPs (27, 28), and that model is generally accepted to be correct.

### RNP architecture-imposed segment condensation.

**FIG 7:**
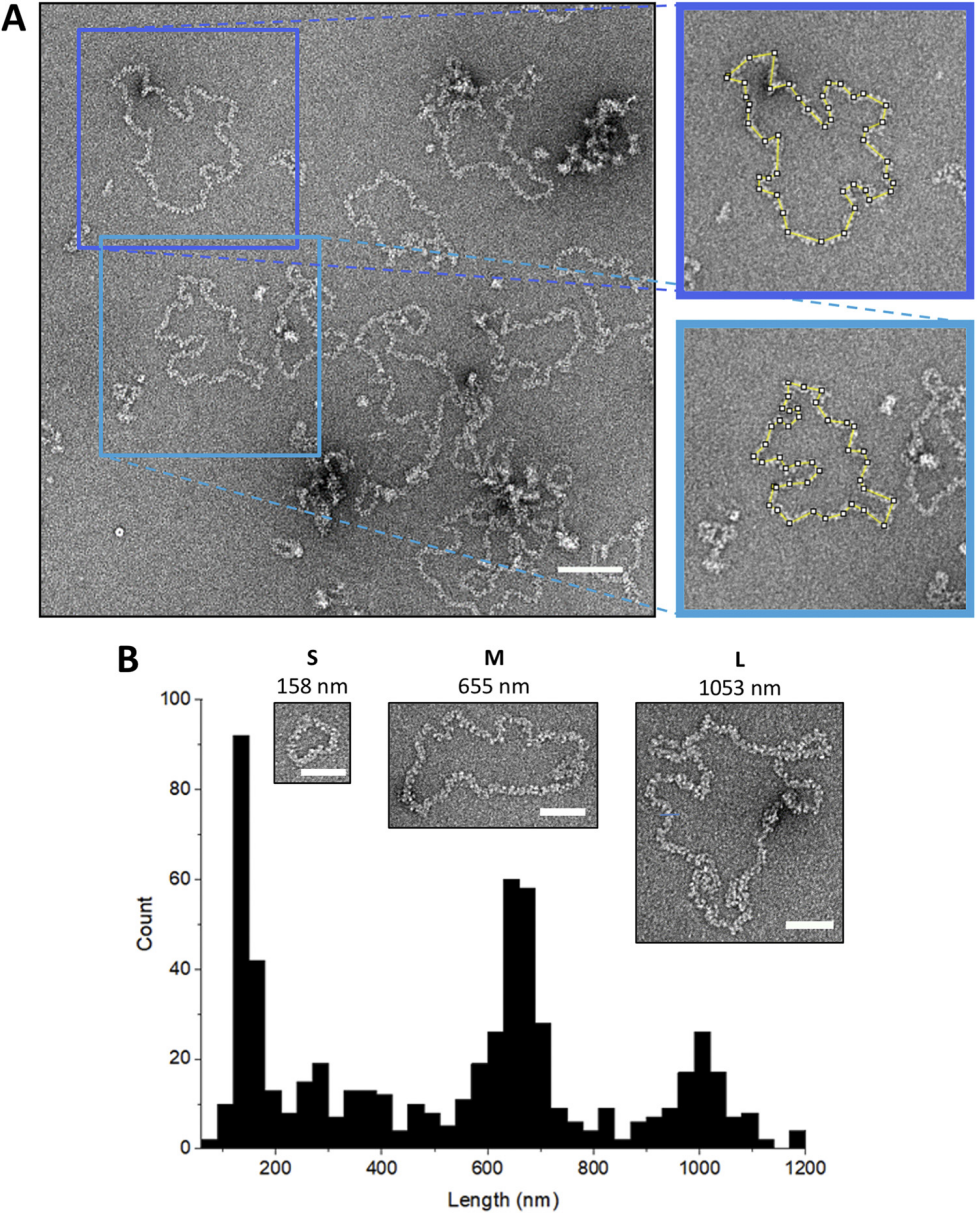
BUNV RNP length measurements. (A) Schematic of discrete RNP tracing. Scale bar: 100 nm. (B) Tracing the lengths of filaments revealed three distinct populations corresponding to S, M, and L genomic segments. Insets show representative S, M, and L RNPs and their lengths. Scale bars, 50 nm.

To gain further insight into the organization of the BUNV RNPs, we measured their overall lengths. Given the nucleotide lengths of BUNV RNA segments (961 nt for S; 4458 nt for M, and 6875 nt for L), and that the orthobunyaviral NP is approximately 5 nm wide and bound approximately 11 RNA bases (12–16, 29), the ‘beads on a string’ organization would predict their lengths to be around 430, 2020 and 3125 nm, respectively. Approximately 600 discrete, circularized RNPs were manually traced and measured (Fig. 7A and B), with the majority falling into three distinct species with mean lengths of approximately 160, 650, and 1050 nm, suggesting a consistent degree of condensation and discarding of the ‘beads on a string’ as a possible arrangement of BUNV RNPs (Fig. 7B). Assuming a helical NP arrangement, the helical rise was estimated to be ~1.7 nm by dividing the RNP lengths by the estimated number of NP monomers per segment based on the NP:nucleotide stoichiometry.

### BUNV RNPs have a helical organization.

**FIG 8:**
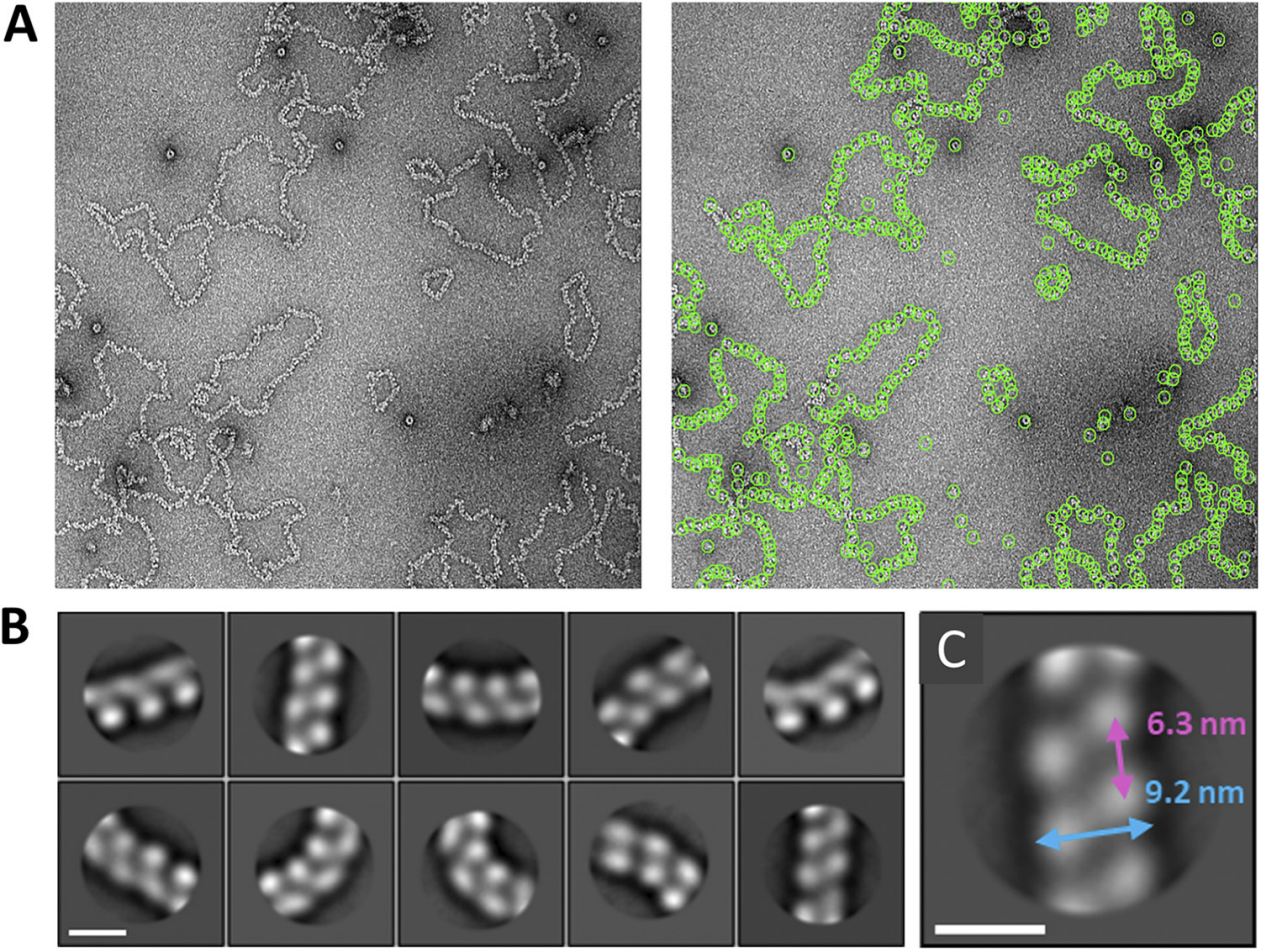
Negative staining EM and 2D classification of BUNV RNPs. (A) Representative micrograph showing abundant, purified BUNV RNPs (left), and the effectiveness of the Relion autopicking tool for selecting particles (right). (B) Alignment and classification clearly showed helix-like architecture within filaments. Scale bar, 10 nm. (C) Estimation of the RNP width and helical pitch. Scale bar, 10 nm.

Alignment and 2D classification of particles picked along the NP portion of the RNPs were used to further elucidate the architecture of the filament. Because RNP filaments exhibited a high degree of flexibility, which allowed them to bend up to ~90^°^ angles, particles were picked with a relatively small box size of 294 Å (Fig. 8A). The alignment and 2D classification of these particles clearly showed a pattern of alternating NP monomers along the filament length, consistent with a helical structure (Fig. 8B). Analysis of straight class averages allowed measurement of the width of the filament and of the distance between monomers along the length of the filament, which in principle represents an approximation of the helical pitch. Measuring three points (center and each end) along the filament in the 10 class averages (Fig. 8B) resulted in mean values of 9.2 nm for the width and 6.3 nm for the longitudinal distance between monomers (Fig. 8C). This corresponded to ~3.7 monomers per helical turn (6.3 nm helical pitch over 1.7 nm helical rise, as determined above). We note that the value for the helical pitch varied from 4.9 to 8.6 nm, in agreement with the observation that the distances between individual monomers could vary to accommodate bends in the filament. Assuming that the helical portion of the RNPs had 3.7 NP per turn, 11 bases per NP, and a 6.3 nm pitch, the lengths of S, M, and L segments (nucleotides per segment/[3.7×11]×6.3) would be 149, 690 and 1064 nm, respectively, very close to the measured mean lengths of 160, 650 and 1050 nm (Fig. 7). This calculation validates the correct identification of the S, M, and L segments, that they were helical, and that the helical parameters we obtained are close to the correct ones.

### Atomic force microscopy (AFM) and STA confirmed that BUNV RNPs were helical.

**FIG 9:**
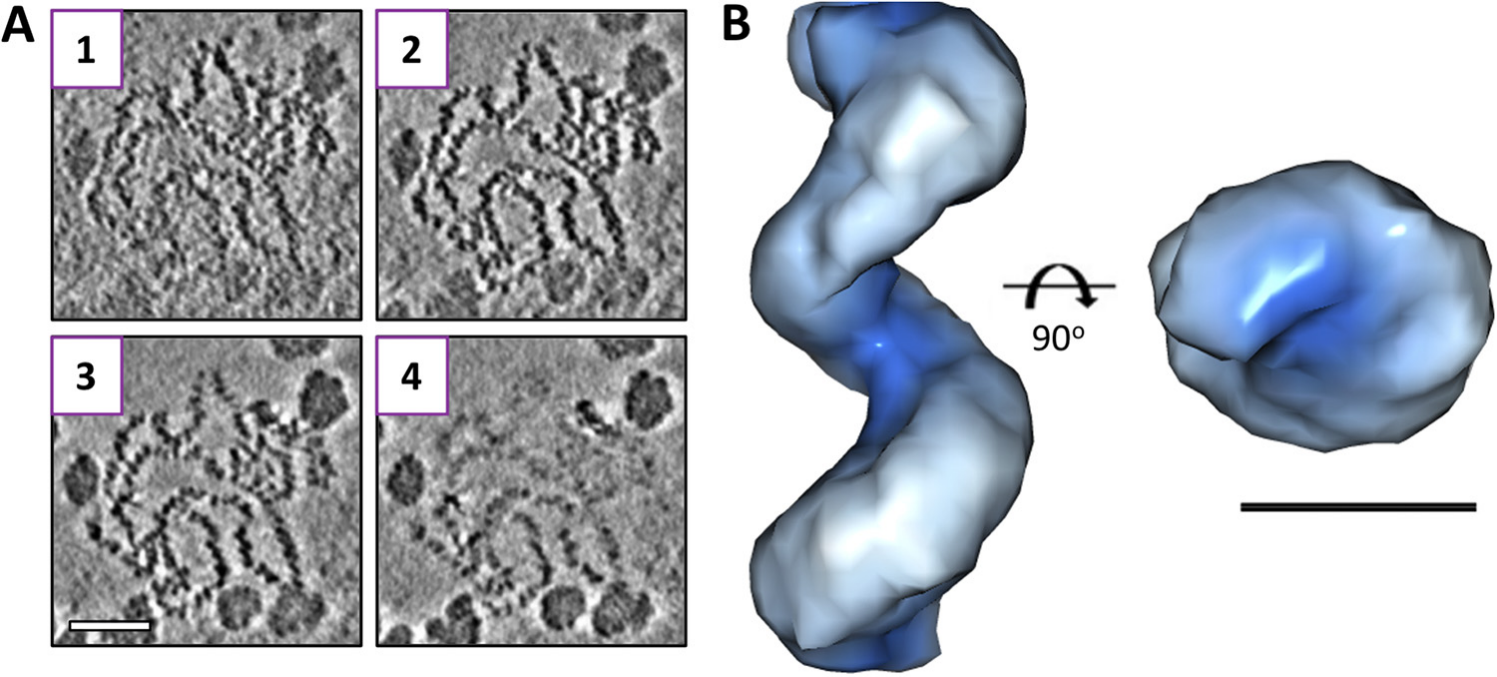
Cryo-ET derived model of a BUNV RNP. (A) Reconstructed tilt-series revealed clear RNPs and numerous electron-dense particles. Insets 1 to 4 show slices 13.5 Å apart within an area containing RNP(s). Scale bar, 50 nm. (B) Subtomogram averaging produced a 20 Å reconstruction of a helical BUNV RNP. Scale bar, 5 nm.

**FIG 10:**
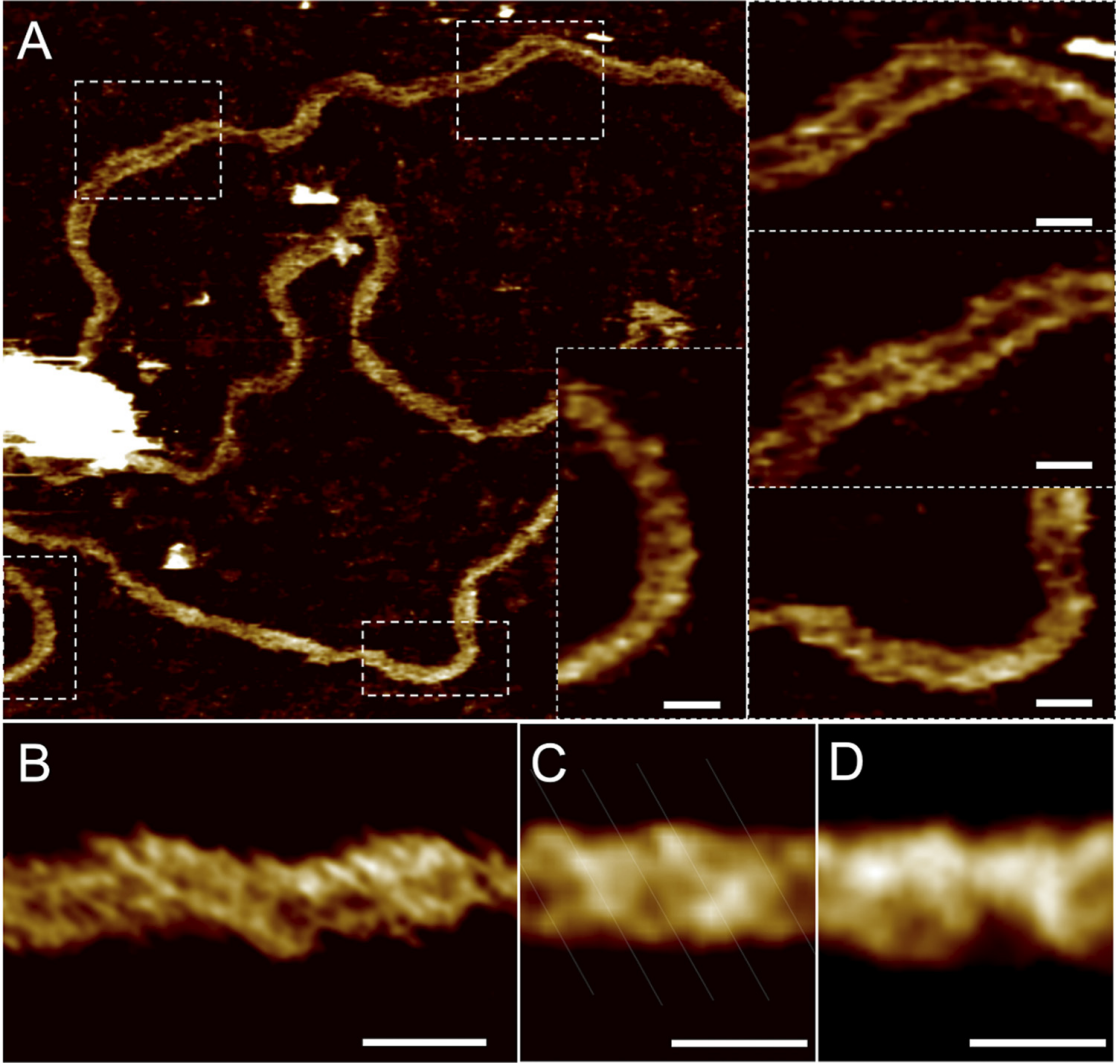
AFM imaging of BUNV RNPs on mica in liquid. (A) AFM image of RNP with digital zooms displaying helical features. (B) High-resolution AFM image of RNP. (C) Correlation average along the length of a digitally straightened protein using a 20 nm reference section of the RNP in (A) (*n* = 80). Lines highlight the right-handedness of the average. (D) Correlation average using the 20 nm reference section with Y-inverted (*n* = 80). All scale bars: 10 nm.

To further confirm that BUNV RNPs were helical, we employed cryo-ET and STA. The use of a Volta phase plate during data collection resulted in high-contrast tomograms in which clear RNP filaments were apparent (Fig. 9A). Additionally, large electron-dense particles not present in the negative staining preparations were observed that possibly represented condensed RNPs within the air-water interface but did not obstruct the reconstruction of tomograms of the subtomogram selection along the RNP lengths. STA of the helical portion of the RNPs was performed using a cylindrical reference and a 20 Å average was produced, which displayed the helical nature of the filament with a width of ~76 Å and helical pitch of ~65 Å (Fig. 9B and Fig. S1). Because EM data does not have a definitive handedness (30), we imaged the RNPs using atomic force microscopy (AFM) (Fig. 10), which can be used to determine the handedness of helical assemblies (31). Standard and high-resolution AFM showed clear right-handed RNP sections (Fig. 10A inset, and Fig. 10B). The handedness of the RNPs was more apparent when averaging NP sections using a right-handed reference (Fig. 10C). Of note, the average was also right-handed when using a reference with an inverted hand (Fig. 10D). Therefore, we concluded that BUNV RNP helices are right-handed.

10.1128/mbio.01405-22.1 FIG S1 FSC curve of the subtomogram average. Resolution is 20 Å at an FSC cutoff of 0.5. Download FIG S1, PDF file, 0.07 MB. Copyright © 2022 Hopkins et al. 2022 Hopkins et al. https://creativecommons.org/licenses/by/4.0/ This content is distributed under the terms of the Creative Commons Attribution 4.0 International license.

### Cryo-electron microscopy produced a 13 Å average of a native BUNV RNP.

**FIG 11:**
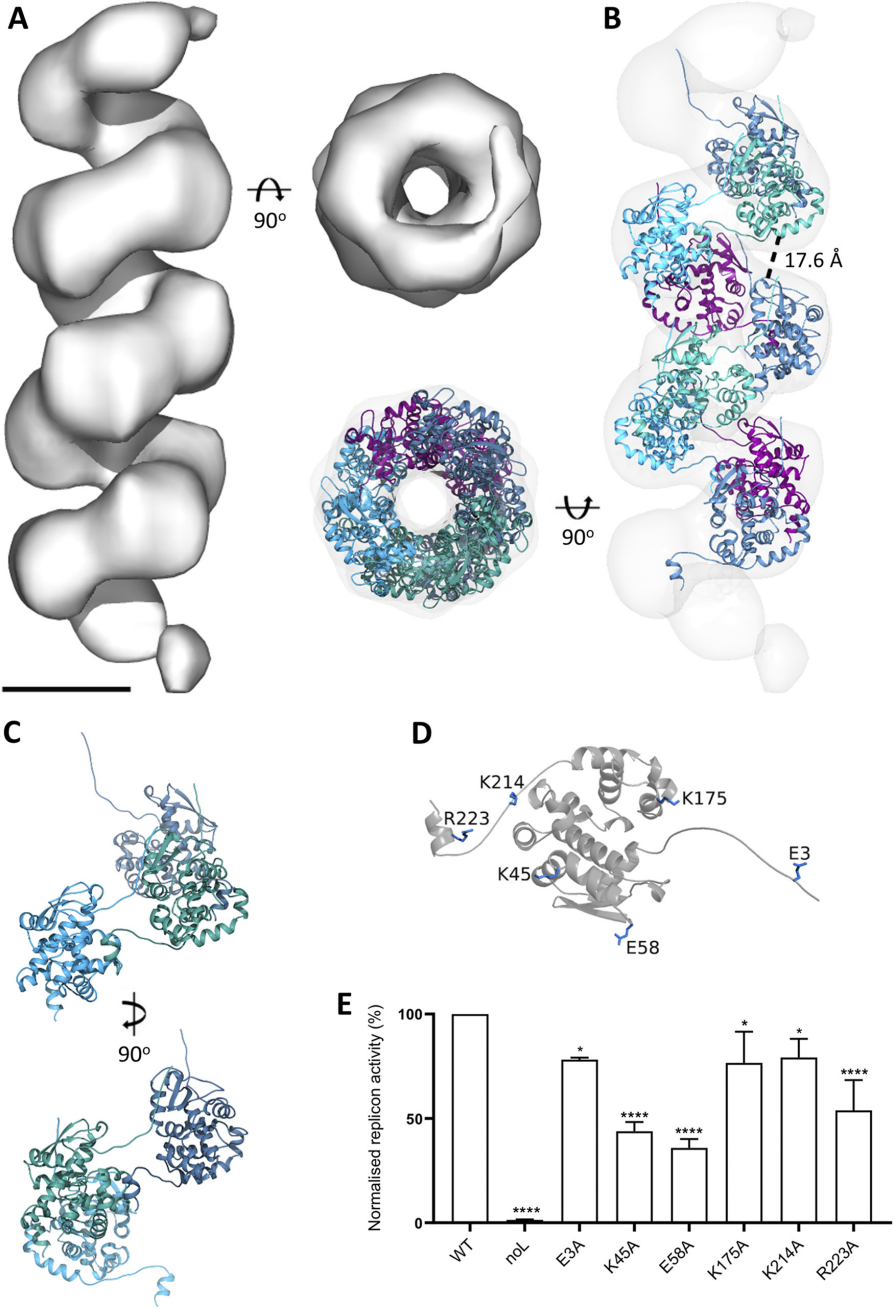
Cryo-EM and pseudo-atomic model of the BUNV RNP. (A) Cryo-EM reconstruction with helical symmetry generated a 13 Å average of a BUNV RNP filament. Scale bar: 5 nm. (B) Flexible fitting of a ‘split’ NP model with appropriate restraints produced a pseudo-atomic model of the BUNV RNP. Distance between rungs of the helix indicated. (C) Orientation of NP within the BUNV RNP model. (D) Tested mutants highlighted on an NP monomer from the RNP helical model. (E) Histogram showing gene expression activity of mini-genome RNPs reconstituted using WT and mutant NPs, alongside transfections in which the BUNV polymerase was omitted (noL). *, *P* ≤ 0.05; **, *P* ≤ 0.01; ***, *P* ≤ 0.001; ****, *P* ≤ 0.0001. Experiments were performed in triplicate.

A cryo-EM data set was then collected with the Volta phase to increase contrast and aid in particle selection (Fig. S2). Given the absence of long, straight NP sections amenable to helical reconstruction, ~135,000 short NP sections were selected and processed using a single particle approach. To validate the helical nature of the RNP, these particles were first subjected to 3D classification using a cylinder as a reference (removing any possible bias toward a helical model). The resulting averages were helical (Fig. S3). Therefore, the particles were taken forward for image processing using the STA-derived model as a reference. Following a similar approach as described recently for the influenza virus (28), which involved classifying particles into small sets of ~4,000 homogeneous particles, we obtained a 14 Å resolution average without symmetry applied (Fig. S4A and B). When applying helical symmetry to this average, with searches centered on −100^°^ for twist and 15 Å for the rise, the searches routinely converged on −104.9^°^ twist and 17.7 Å rise. Therefore, these numbers were applied for a final reconstruction with local searches, resulting in a 13 Å average that converged on a −105.26° twist and 18.25 Å rise (Fig. 11A and Fig. S4C). These helical parameters agreed with our estimated parameters from negative staining (3.4 NPs per turn and 18.25 Å rise versus the estimated 3.7 NPs per turn and 17 Å rise). On average, the helix was not tightly wound or compact.

10.1128/mbio.01405-22.2 FIG S2 Schematic illustration of the image processing of cryo-EM data. Blue volumes denote the classes taken forward to further processing. Download FIG S2, PDF file, 1.0 MB. Copyright © 2022 Hopkins et al. 2022 Hopkins et al. https://creativecommons.org/licenses/by/4.0/ This content is distributed under the terms of the Creative Commons Attribution 4.0 International license.

10.1128/mbio.01405-22.3 FIG S3 Unbiased processing of BUNV RNP cryo-EM data. Classification with a cylinder reference showed a clear helix in the absence of any symmetry or reference bias. Download FIG S3, PDF file, 0.7 MB. Copyright © 2022 Hopkins et al. 2022 Hopkins et al. https://creativecommons.org/licenses/by/4.0/ This content is distributed under the terms of the Creative Commons Attribution 4.0 International license.

10.1128/mbio.01405-22.4 FIG S4 BUNV RNP cryo-EM averages. (A) Cryo-EM average of BUNV RNPs without applying helical symmetry. FSC curves of (B) the asymmetric average and (C) the symmetric average. FSC cutoff 0.143. Download FIG S4, PDF file, 0.9 MB. Copyright © 2022 Hopkins et al. 2022 Hopkins et al. https://creativecommons.org/licenses/by/4.0/ This content is distributed under the terms of the Creative Commons Attribution 4.0 International license.

### NP crystal structures and molecular dynamics allowed the generation of an atomic model for the NP portion of BUNV RNP.

The generation of a 3D helical model of the NP portion of BUNV RNP permitted the fitting of a ‘split’ NP crystal structure (Fig. S5) to generate a pseudo-atomic model. Briefly, in all available crystal structures of multimeric orthobunyavirus NP, the globular core of the protein is highly conserved (Fig. S5A), as are the binding positions of the N- and C-terminal arms relative to their bound neighbor (Fig. S5B). To preserve these aspects of the structure, we produced a model of what we termed ‘split’ NP (Fig. S5C to E) that contained the core of a single NP monomer and the bound arms of its two neighbors. A limitation of this approach was that it prevented us from modeling the viral RNA along the NP arms, and therefore, we did not include it in our model. The NP structure was symmetrized, and molecular dynamics were applied to restrain the ‘split’ NP. This allowed freedom of movement of the linker region of each terminal arm, which had already been shown to be flexible while minimizing alterations to the globular core of the protein or the binding regions of the arms (Fig. S5A). As a result, we obtained a helical NP model for BUNV RNP (Fig. 11B and C). Displaying the surface coulombic potential of the helical model confirms that the positively charged RNA-binding groove of each monomer is aligned in a way that maintains an RNA-binding channel along with the interior of the entire helical filament (Fig. S6A). Studying the surface coulombic potential also ruled out the possibility of longitudinal electrostatic interactions between charged areas on the surface of monomers because the surfaces of the protein that face each other longitudinally contained mainly negatively charged residues (Fig. S6B). Furthermore, these rungs were too far apart (approximately 18 Å) to permit intermolecular interactions of any kind between NP monomers along the longitudinal axis (Fig. 11B).

10.1128/mbio.01405-22.5 FIG S5 Generation of a ‘split’ BUNV NP for fitting NP to the BUNV RNP model. (A) Alignment of available full NP structures from different orthobunyaviruses. Our RNP-derived model is in blue. The arms are flexible. (B) Alignment of ‘split’ NPs from the same orthobunyaviruses as in panel A. Our RNP-derived model is in pink. PDB accession codes: 3ZLA, 3ZL9, 4BHH, 4IJS, 4J1J, 4JNG. (C) Illustration of the arrangement of NP within an available tetrameric crystal structure. (D) A full NP molecule within the crystal structure with the core of NP^B^ in purple and both arms of NP^B^ in red. (E) A ‘split’ NP, comprising the core of NP^B^ (purple) and the arms of neighboring NP^A^ and NP^C^ (red). Download FIG S5, PDF file, 0.7 MB. Copyright © 2022 Hopkins et al. 2022 Hopkins et al. https://creativecommons.org/licenses/by/4.0/ This content is distributed under the terms of the Creative Commons Attribution 4.0 International license.

10.1128/mbio.01405-22.6 FIG S6 Coulombic surface potential of the helical BUNV NP model. (A) The BUNV RNP model has a continuous positively charged RNA-binding groove pointing towards the inside of the RNP (blue). (B) Coulombic surface potential shows electrostatic interactions between lateral neighboring NP monomers are unlikely. Download FIG S6, PDF file, 0.5 MB. Copyright © 2022 Hopkins et al. 2022 Hopkins et al. https://creativecommons.org/licenses/by/4.0/ This content is distributed under the terms of the Creative Commons Attribution 4.0 International license.

### Using the native RNP model to identify NP residues involved in the formation of active RNPs.

The generation of our helical NP model allowed investigation of the mechanism of native RNP assembly, in terms of the formation of both the NP-NP multimer chain, and its helical architecture. The previous interrogation of the BUNV NP crystallographic model (12) revealed NP-NP contacts responsible for driving the formation of the closed tetrameric ring. However, by comparing these NP-NP contacts with those within the native helical NP, 7 novel residues were identified between laterally adjacent NP monomers with sidechains potentially involved in weak H-bond or salt bridge interactions within the RNP helix (Tables S1 to S3 and Fig. 11D). Because the resolution of our map prevented us from refining the side chains of NP, to test their involvement in helical NP formation, each of these 7 residues was individually substituted for alanine in reconstituted RNPs, and their resulting function was tested by mini-genome analysis using reporter gene expression as a measurement of active RNP assembly. First, we tested if the mutations perturbed NP expression. All mutants except E173A resulted in a level of expression comparable to that of wild-type NP (Fig. S7). The ability of the remaining 6 NP mutants to support active RNP formation was tested using a mini-genome assay, with all resulting in a significant reduction of reporter activity (Fig. 11E). This finding is consistent with each of these residues playing roles in the formation of an active RNP and thus further validates our helical NP model.

10.1128/mbio.01405-22.7 FIG S7 Comparison of expression of mutant compared to wild type NP. (A) Western blot with anti-NP and anti-GAPDH of cell lysates after transfecting the BUNV mini-genome system, with the specified mutations. (B) The ratio of net BUNV NP to GAPDH was measured by densitometry. ns, *P* > 0.85; *, *P* ≤ 0.05; **, *P* ≤ 0.01; ***, *P* ≤ 0.001; ****, *P* ≤ 0.0001. Experiments were performed in triplicate. Download FIG S7, PDF file, 0.2 MB. Copyright © 2022 Hopkins et al. 2022 Hopkins et al. https://creativecommons.org/licenses/by/4.0/ This content is distributed under the terms of the Creative Commons Attribution 4.0 International license.

10.1128/mbio.01405-22.8 TABLE S1 Helical model NP-NP interactions as defined by PDBePISA. Download Table S1, PDF file, 0.04 MB. Copyright © 2022 Hopkins et al. 2022 Hopkins et al. https://creativecommons.org/licenses/by/4.0/ This content is distributed under the terms of the Creative Commons Attribution 4.0 International license.

### The contribution of RNA to maintaining the RNP architecture.

**FIG 12:**
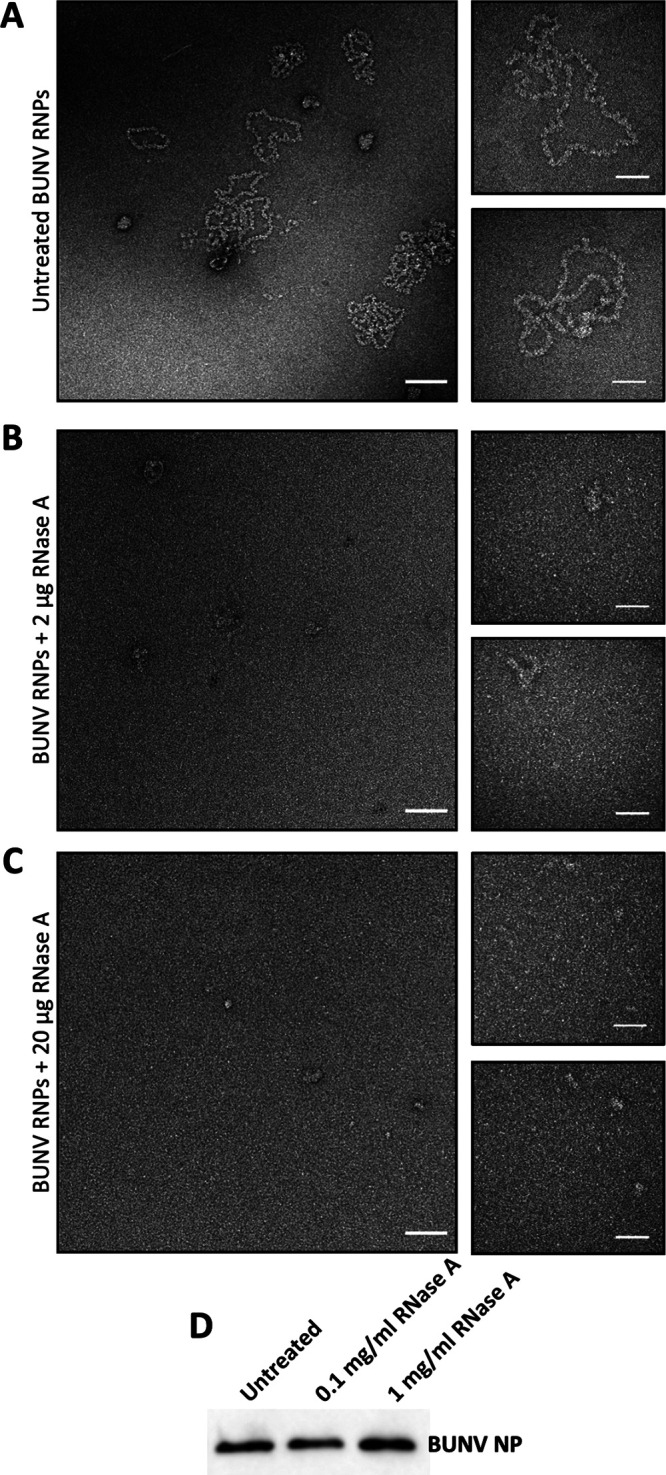
The RNA component of BUNV RNPs contributes to maintaining the helical arrangement. Purified RNPs were treated with different amounts of RNase A and visualized using negative staining EM. Representative micrographs of (A) Untreated BUNV RNPs, (B) BUNV RNPs treated with 2 μg of RNase A (0.1 mg/mL), and (C) BUNV RNPs treated with 20 μg of RNase A (1 mg/mL). Scale bars: 100 nm in main and 50 nm in insets. (D) Western blotting against BUNV NP of the different samples imaged by negative stain EM.

To test whether NP-NP interactions are sufficient alone to maintain RNP integrity, purified BUNV RNPs were incubated with RNase A at concentrations of 0.1 mg/mL and 1 mg/mL, known to degrade BUNV NP-encapsidated RNA (29), and then visualized by negative staining EM. Characteristic BUNV RNPs were abundant in untreated samples (Fig. 4, 7, 8, and 12A), whereas following RNase treatment, no RNPs were detected (Fig. 12B and C). Instead, a diffuse background staining was apparent, most likely comprising dissociated NP monomers not observed in untreated samples. Of note, intact NP monomers were present in all these samples, as assessed by western blot (Fig. 12D), suggesting that NP-NP dissociation and not protein degradation was responsible for the loss of intact RNPs. This finding suggests that the encapsidated RNA is important for the integrity of the orthobunyaviral RNP.

## DISCUSSION

We have studied native orthobunyaviral RNPs using a combination of microscopy approaches. Our current and previous EM observations show that the orthobunyavirus RNP exhibits a high degree of flexibility and thus heterogeneity, which has hampered the application of techniques to derive high-resolution structural models of RNPs from this group of viruses. For example, while we generated a medium-resolution cryo-EM average of the NP portion of native purified BUNV RNPs, fitting the structure of the NPs within the RNP helix was hindered due to the resolution. Therefore, we combined restraints derived from the analysis of deposited orthobunyavirus atomic models of NP oligomers with molecular dynamics to obtain a pseudo-atomic model of the helical NP of BUNV RNPs. Our data conclusively shows that the three segments of BUNV RNP present a consistent degree of condensation and that they adopt a helical conformation yet were highly flexible. Furthermore, we show that the stability of the RNPs is dependent on the integrity of the RNA, providing key information about the RNP assembly and structure.

RNP helicity is a universal feature of negative-sense RNA viruses. For the nonsegmented negative sense (NSNS) RNA viruses, the RNP helices are maintained through both lateral and longitudinal NP-NP interactions, which result in high levels of compaction, and in rigid RNPs, exemplified by measles and Ebola viruses (32–36). The parameters of high levels of compaction and high rigidity for NSNS RNPs are in stark contrast to those of BUNV RNPs, and it is interesting to speculate why this might be. In terms of compaction, we showed the rather loose helicity of the BUNV RNP results in a condensation of each segment length by approximately 3-fold. However, the calculation of the internal volume of BUNV particles, given an average diameter of around 108 nm (11), suggests an internal volume of around 6.7 × 10^5^ nm^3^. By treating the three BUNV RNPs as a single cylindrical volume with a diameter of 9.2 nm (Fig. 8C) and a combined length of 1850 nm, the volume that the three segments would occupy according to our RNP model is 1.2 × 10^5^ nm^3^, approximately one-fifth of the capacity of the virion. From these calculations, we propose that further RNP compaction is not necessary for genome incorporation within virions of average dimensions. An alternative factor that might have driven the evolution of BUNV RNP helicity is an evasion of RNA insult and innate immune detection. A helix effectively represents an enclosed tube, and in the case of an NSNS RNA virus RNP, one that can offer a high level of RNA protection. However, BUNV RNPs only possess partial RNase resistance; we hypothesize that this is due to the requirement of BUNV RNPs to be flexible, which is a trade-off for a highly compacted highly protected RNP. Additionally, we note that the purification process and the buffer conditions might influence the RNase susceptibility of the RNP and that the conditions within an infected cell might be different than *in vitro*. Therefore, the RNA accessibility within the RNP warrants more investigation.

In the case of BUNV RNPs, the level of flexibility observed in the native helical BUNV RNPs permits a large degree of bending (up to 90^°^; e.g., Fig. 4B) while still apparently preserving the integrity of the filament, which is likely of great benefit for several stages of the replication cycle. Similar to condensation, described above, RNP flexibility will facilitate segment packaging during virion assembly, as it will allow the genomic segments to contort within the virion interior. Our results showing that the RNP architecture is dependent on the presence of encapsidated RNA hint at a possible explanation for the RNP flexibility. As in the crystallographic atomic models, the viral RNA is protected by the NP in the interior of the rings NP formed when expressed recombinantly (12, 13, 15, 16), we hypothesize that the RNase accessible RNA locates between two adjacent NPs, which would also result in flexible helical RNPs. We note that Ariza et al. (12) reported that recombinantly expressed NPs protect RNA from RNase treatment. However, in that study, RNase was added directly to crude bacterial cell lysates (at 8 μg/mL), whereas in the current manuscript RNase was added to a purified NP-RNA complex (at 0.1 and 1 mg/mL), and thus the outcomes are not comparable.

Previous mini-genome mutagenesis suggests that 5′ and 3′ terminal sequences functionally interact with each other to promote BUNV gene expression (37–39). Furthermore, structural analysis reveals that RdRp possesses binding sites for both segment ends (24, 40). While both these observations are consistent with segment circularization, whether circularization is needed for RdRp to bind, or because the RdRp protein binds specifically to both ends, is currently unclear. In any case, we hypothesize that RNP flexibility also allows the BUNV segments to adopt their characteristic circular RNP conformation in which their 3′ and 5′ ends interact, and this ability is particularly important for the S segment, for which circularization requires the shortest radius of curvature due to its short nucleotide length. Likely related to the trade-off between flexibility and compaction/protection, RNP circularization does not appear to be a requirement of NSNS RNA viral genomes, which may explain their adoption of a more compacted, rigid RNP architecture. It is interesting to note that the RNP architecture adopted by the segmented influenza virus (27, 28, 41) provides a high degree of compaction, mediated by lateral and longitudinal NP-NP contacts, while at the same time allowing terminal interactions necessary for RNA synthesis. The solution to this structural paradox has been to restrict RNP flexibility to a short mid-segment loop, allowing the path of the RNP to make a tight 180^°^ turn, allowing the formation of an anti-parallel double-helical rod-like assembly that positions the termini side-by-side.

The current model for how native orthobunyaviral RNPs circularize is unclear, with possible contributions from interterminal Watson-Crick base-pairing interactions, and interactions with sequence-specific binding sites on the RdRp surface. Our negative staining EM observations revealed BUNV RNPs to be almost exclusively circular molecules, with less than 1% appearing in a linear form. Interestingly, close inspection of the circular RNPs revealed no evidence of where the joins between the RNA 3′ and 5′ termini might be. This finding was unexpected, as the interterminal interactions shown to be important for BUNV RNA synthesis (37–39) would be predicted to result in the formation of a partial duplex by Watson-Crick pairings, distinct from all other segment sequences that are single-stranded. Because the NP RNA-binding groove cannot accommodate double-stranded RNA (12–16), this terminal hairpin could be predicted to be displaced from the oligomeric NP chain, resulting in a region of the RNP with a distinctive structure. We were unable to detect any such regions, although this may reflect the relatively low resolution of negative staining EM.

An alternative mechanism for segment circularization is that it is mediated by the RdRp, based on the observation that the 3′ and 5′ terminal sequences bind to separate sites on the RdRp surface (24, 40). However, most of the RNPs we imaged lacked a putative RdRp, which leads us to speculate that RNP circularization is not solely dependent on the presence of a resident RdRp to tether the segment termini. Circularization independent of the presence of an RdRp raises the possibility that interactions between adjacent NP molecules alone may drive segment circularization, leading to the formation of a continuous NP chain without distinctive ends. However, complementary ends are critical for the viral life cycle. Therefore, the genome ends must interact at some point in the infectious cycle. It is of note that native RdRp are not routinely observed in association with their corresponding bunyaviral RNPs (12–15), in contrast to that influenza virus for which the RdRp readily copurifies as part of the RNP complex (27, 28). This suggests that while the bunyavirus RdRp has an overall similar architecture to the influenza virus polymerase complex (24), their properties are very different; it also might suggest weak RdRp interactions within bunyavirus RNPs, although we cannot rule out that the disparity might also be caused by the different RNP purification protocols.

The flexible helical arrangement of BUNV RNPs contrasts with the recently solved structure of an *in vitro* reconstituted Hantaan virus RNP (23), which appears as a rigid helix. This finding, along with significant differences in NP structure and multimerization strategies across the five animal and human infecting families that make up the *Bunyavirales* order suggests their corresponding RNPs may also show significant differences in the overall architecture, with potential implications for the design of future therapeutics targeting RNPs from this order of viruses.

## MATERIALS AND METHODS

### Virus propagation and purification.

BUNV was propagated in BHK-21 cells and purified as described previously (42, 43). Briefly, baby hamster kidney (BHK-21) cells at 80 to 90% confluence were infected with BUNV at a multiplicity of infection (MOI) of 0.01 and maintained in serum-free DMEM at 32°C. After 96 h supernatant was collected and clarified by centrifugation at 3,700 × *g* for 20 min. Virus particles were then purified and concentrated by centrifuging at 100,000 × *g* for 3 h in an SW32 rotor (Beckman-Coulter) with an underlay of 30% d-sucrose prepared in TNE buffer (100 mM Tris-HCl, 200 mM NaCl, 0.1 mM EDTA, pH 7.4) supplemented with protease inhibitors (Roche). Pellets were resuspended in 100 μL of TNE buffer (200 mM NaCl) at 4°C overnight and pooled the following day.

### RNP purification.

RNPs were purified based on a previous protocol (12). Virions were disrupted by a single freeze/thaw cycle, 30 min incubation in 1% Triton X-100 (Sigma-Aldrich) and 0.1% NP-40 alternative (Calbiochem) or 30 min incubation in 30% d-sucrose in TNE buffer. Disrupted virions were applied to the top of a continuous 10 to 25% OptiPrep gradient prepared in TNE buffer (200 mM NaCl; described above) and centrifuged in an SW60 rotor (Beckman-Coulter) at 250,000 × *g* for 90 min. Fractions were collected and analyzed for the presence of BUNV NP by western blotting using specific antisera (produced in-house (44)), and those with the strongest NP signal (corresponding to approximately 23% OptiPrep) were buffer-exchanged to remove OptiPrep.

### Negative staining EM.

Immediately following buffer exchange into reduced-salt TNE (25 mM NaCl), 5 μL aliquots of RNPs were adsorbed to glow-discharged, carbon-coated copper grids for 3 min. Following two water washes, grids were stained for 30 s with 1% uranyl acetate. Images were collected on a 120 kV FEI Tecnai G^2^-Spirit microscope at a nominal magnification of 45,000×.

The NP portion of BUNV RNPs was subject to autopicking in Relion 3.0 (45) and a total of 175,583 particles were extracted. Rounds of 2D classification were used to discard the most heterogeneous particles and a final set of 14,899 particles was used to generate 2D class averages. To estimate the RNP length, 609 discrete RNP filaments were manually traced in Fiji (46) using the line tool, and the length of the line was determined and used as an estimation of RNP length. Similarly, the line tool was also used to measure filament diameters and the distance between neighboring subunits from within-class averages.

### Cryo-ET and STA.

Carbon-coated quantifoil grids were prepared by plunge-freezing in liquid ethane using an FEI Vitrobot IV. Cryo-ET tilt-series were collected on an FEI Titan Krios EM 300 kV microscope equipped with energy-filtered Gatan K2 XP Summit and Volta phase plate (Astbury Biostructure Laboratory, University of Leeds). Tilt-series were collected at 2^°^ intervals from −60^°^ to 60^°^ at a nominal magnification of 71,000× for a pixel size of 2.7 Å. During tilt series and single-particle data collection, on-the-fly motion correction and contrast transfer function (CTF) estimation were set up in Relion 3.0 as previously described (47). Tomograms were reconstructed within the eTomo pipeline for fiducial-less alignment using weighted back-projection from the IMOD software package (48). STA was carried out using PEET (49). Subtomograms of the NP portion of BUNV RNPs were manually picked using 3dmod and STA was performed by alignment and averaging of these subvolumes in PEET, using the absolute value of cross-correlation and strict search limit checking. For the first iterations, only the orientation of the subtomograms (without rotation) was included in the angular search range, starting with a maximum of 24^°^ and a step of 8^°^ and halving with each iteration, to produce a straight cylindrical model. In later iterations when the subtomograms were orientated down to 6^°^, the rotation search was included. Approximately 2,000 subtomograms were included in the final average resulting in a resolution of 20 Å (estimated by Fourier shell correlation at a 0.5 cutoff), which was visualized using University of California San Francisco (UCSF) Chimera (50).

### AFM sample preparation and imaging.

The handedness of the NP portion of BUNV RNPs was determined using AFM. Eighteen microliters of RNPs were deposited on freshly cleaved mica in an imaging buffer of 10 mM NiCl_2_, 10 mM HEPES pH 7.5. All AFM imaging was carried out at room temperature with the RNPs in solution and hydrated in the imaging buffer. AFM observations were performed in tapping mode using a Dimension FastScan Bio with FastScan-d-SS probes (Bruker). The force applied by the tip on the sample was minimized by maximizing the set point while maintaining tracking of the surface. Images were processed using first-order line-by-line flattening to remove the sample tilt and background using Nanoscope Analysis (Bruker). Correlation averaging was performed using self-written code in MATLAB. RNPs were first skeletonized to trace the profile before profile smoothing and digitally straightening. A reference NP section within the digitally straightened image containing 1 full helical repeat of the RNP, was then cross-correlated along the entire length of protein to search for similar features. Correlation averages were created using the 80 of 120 sections with the highest cross-correlation coefficient. Cross-correlation was also performed with a vertically flipped reference image to search for possible left-handedness. The code employed for AFM image analysis has been deposited in https://github.com/George-R-Heath/Correlate-Filaments.

### Cryo-electron microscopy.

Grids for cryo-EM were prepared as for cryo-ET. Single-particle data were collected on an FEI Titan Krios microscope operating at 300 kV and equipped with a Falcon 3EC. Micrographs were collected at a nominal magnification of 130,000× for a pixel size of 1.07 Å.

Data were processed in Relion 3.0 (45). Approximately 1,000 particles were picked for reference-free classification and the resulting classes were used as the templates for autopicking. A total of 1.47 million particles were selected by autopicking and extracted with a box size of 274 Å, binning particles 4 times (resulting in pixel size 4.28 Å). Two rounds of 2D classification selected the most homogenous 135,102 particles. To validate the helical nature of the NP portion of the RNP, these particles were first subjected to 3D classification using a cylinder as a reference (removing any possible bias toward a helical model). The resulting averages were helical, and therefore the particles were taken forward for image processing using the STA-derived model. The selected particles were 3D classified against the STA model and two further rounds of 3D classification left the 42,237 most homogenous particles. These particles were re-extracted with a smaller box size of 214 Å to increase homogeneity and were subject to two more rounds of 3D classification. In the final round of 3D classification, the particles were divided into classes of approximately 4,000 particles, as was recently done for IAV (28), and the top class was subject to helical reconstruction without symmetry. This resulted in 5,077 particles, which were then re-extracted with a box size of 274 Å to aid in alignment and a final refinement was carried out with helical symmetry applied. Local searches were applied that centered on values of −100^°^ for twist and 15 Å for the rise, as determined by viewing an XYZ projection of the nonsymmetrical model. These always converged on values around −104.9^°^ for twist and 17.7 Å for the rise, so these numbers were used for searches for the final refinement with helical symmetry imposed, which converged on −105.26° twist and 18.25 Å rise. The asymmetric and symmetric reconstructions are highly similar (their cross-correlation value is 0.94 when using the chimera fit in map tool), suggesting we found the correct helical parameters.

### Atomic modeling of BUNV RNPs.

To generate an atomic model of the NP portion of BUNV RNPs, we used the coordinates of BUNV NP as a starting point (PDB: 3ZLA (12)). First, a trimer of NP was rigid-body fitted into the cryo-EM average using Chimera (50). Of the two possible orientations, one of them (which we term ‘beta-strand up’) fit slightly better into the nonhelically symmetrized average (it had an average map value at fit atom positions of 0.09 versus 0.081 for the alternative orientation). However, both orientations were taken forward to allow a comparison of the result. As previously reported, the core domain of all atomic coordinates from orthobunyavirus NP is constant, while the N- and C-terminal arms were flexible (Fig. S5A and (51)). However, when comparing the NP core with the arms of the adjacent subunits (which we termed ‘split’ NP), the interactions between the arms and the core domain remain constant across the structures of all orthobunyavirus NP oligomers (Fig. S5B). Therefore, we next fitted a ‘split’ NP into the central monomer of the rigid-body fitted trimers, and manually added the missing amino acids of the coordinates of BUNV NP in COOT (52) (corresponding to the linker regions between the arms and the core domain), to prevent the movement of the terminal ends of the protein to positions that are not physically possible during flexible-fitting. The resulting NP monomer was helically symmetrized in Chimera, and the NP helix was flexible-fitted into the cryo-EM average using MDFF (53) following the program’s guidelines. The ‘split’ NP domains were kept as rigid domains by applying domain restraints, and symmetry restraints were also employed. Of the two possible orientations of the NP, the ‘beta-strand up’ resulted in a better model, in terms of positive electrostatic charges for interactions with the RNA.

### PISA.

PDBePISA (Protein Interfaces, Surfaces, and Assemblies: http://pdbe.org/pisa/) and Chimera (50) were used to predict interchain interface contact residues, including H-bonds and salt-bridges, involved in the interaction between BUNV NPs within the previously published BUNV NP tetramer (PDB: 3ZLA (12)) and within the helical NP model (Tables S1 to S3).

### BUNV mini-genome plasmids and mutagenesis.

Previously described plasmids were used for the BUNV mini-genome system assay (54). Briefly, the mini-genome system is constituted by BUNV L and NP support plasmids (pT7riboBUNL, pT7riboBUNN), which express BUNV L and NP downstream of the T7 pol promoter and the internal ribosome entry site (IRES) of encephalomyocarditis virus (ECMV), the minigenome pT7riboBUN-SREN, which contains the renilla luciferase gene in the negative sense flanked by the BUNV S segment UTRs, and the control plasmid pCMV-Firefly-Luc, which expresses firefly luciferase under cytomegalovirus (CMV) promoter. Mutagenesis of the NP open reading frame (ORF) was achieved using pT7riboBUNN plasmid as the template for site-directed mutagenesis (SDM). SDM primers were designed using NEBaseChanger and SDM reactions were performed using a Q5 SDM kit (NEB) according to the manufacturer’s instructions. Corresponding DNA clones were transformed into competent bacterial cells, purified using Miniprep kits (Qiagen) and mutations were confirmed using Sanger sequencing.

### BUNV mini-genome transfection and luciferase activity assay.

BSR-T7 cells were seeded in 24-well plates 1 day before transfection at a density of 5 × 10^4^ cells per well. Cells were transfected with 0.2 μg each of pT7riboBUNL, pT7riboBUNN, or one of the mutated NP clones, the mini-genome plasmid pT7riboBUN-SREN and 0.1 μg pCMV-Firefly-Luc (internal transfection control). TransIT-LT1 transfection reagent (Mirus Bio) was used as a transfection reagent in a 2.5:1 ratio of TransIT-LT1 (μL):DNA (μg) according to the manufacturer’s instructions. At 24 h posttransfection, Renilla and firefly luciferase activities were measured using the dual-luciferase assay kit (Promega). Statistical analyses were performed using one-way analysis of variance (ANOVA), followed by Dunnett's multiple-comparison test to determine significant differences in mini-genome activity between NP mutants and WT BUNV NP.

### RNase treatment.

For the RNase treatment, purified BUNV RNPs in TNE were treated with 2 μg of RNase A (resulting in a final RNase concentration of 0.1 mg/mL) or with 20 μg of RNase A (resulting in a final RNase concentration of 1 mg/mL) in a total reaction volume of 20 μL for 30 min at room temperature.

### Data availability.

The asymmetrical and symmetrical averages and pseudo-atomic model are deposited under EMDB accession numbers EMD-11847 and EMD-11849, and PDB accession number 7AOY.

10.1128/mbio.01405-22.9 TABLE S2 Tetrameric model (PDB: 3ZLA) NP-NP interactions as defined by PDBePISA. Download Table S2, PDF file, 0.04 MB. Copyright © 2022 Hopkins et al. 2022 Hopkins et al. https://creativecommons.org/licenses/by/4.0/ This content is distributed under the terms of the Creative Commons Attribution 4.0 International license.

10.1128/mbio.01405-22.10 TABLE S3 BUNV NP-NP interactions exclusive for the helical model as defined by PDBePISA. Highlighted in red: mutants tested in the mini-genome assay. Download Table S3, PDF file, 0.03 MB. Copyright © 2022 Hopkins et al. 2022 Hopkins et al. https://creativecommons.org/licenses/by/4.0/ This content is distributed under the terms of the Creative Commons Attribution 4.0 International license.
