# Supplementary figures and images for: The Native Orthobunyavirus Ribonucleoprotein Possesses a Helical Architecture
Source: mBio. 2022 Jun 28;13(4):e01405-22. doi: 10.1128/mbio.01405-22 (PMC9426602; doi:10.1128/mbio.01405-22)

**Figure S1**

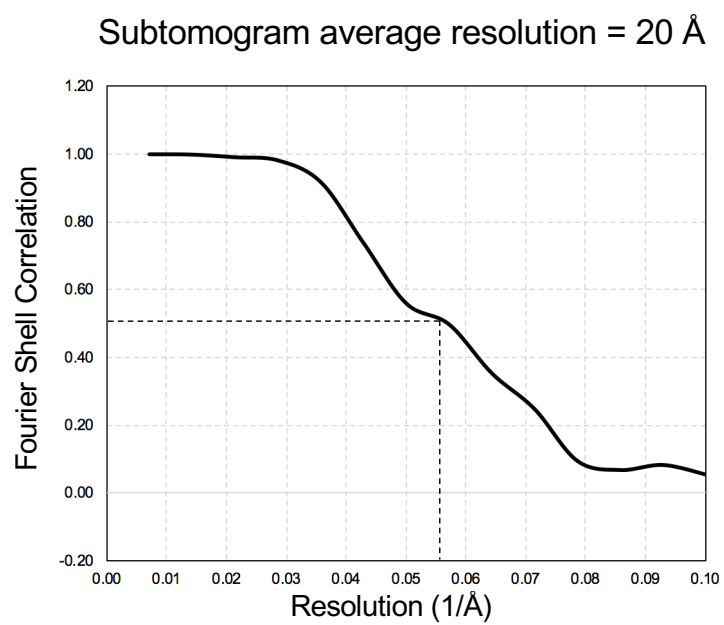

Supplement: FIG S1 [file mbio.01405-22-s0001.pdf]

## Figure S2

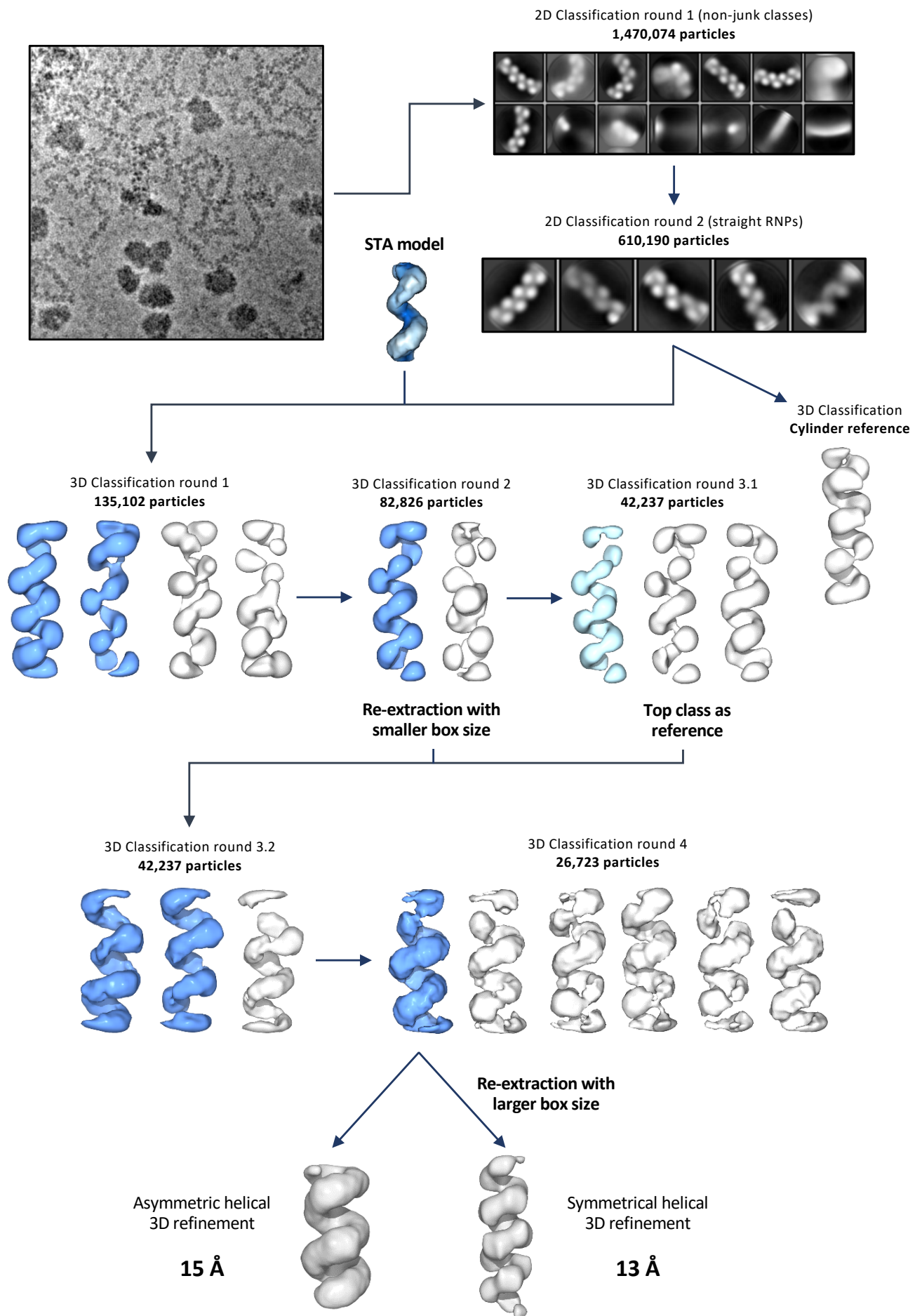

Supplement: FIG S2 [file mbio.01405-22-s0002.pdf]

**Figure S3**

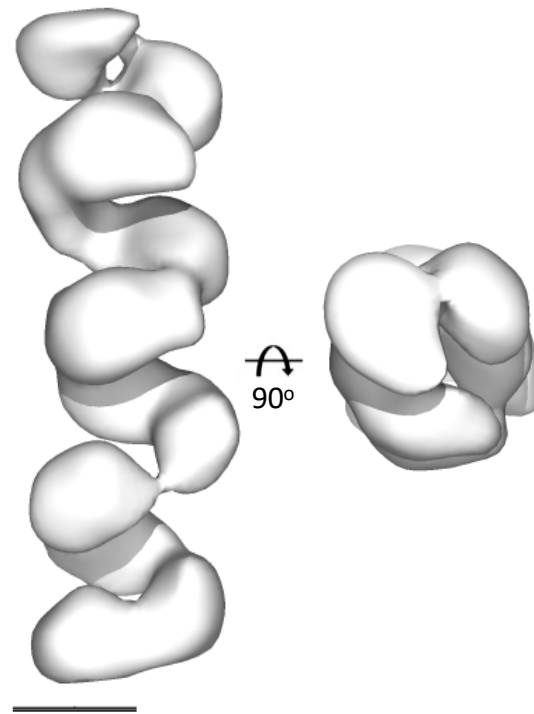

Supplement: FIG S3 [file mbio.01405-22-s0003.pdf]

**Figure S4**

**A**

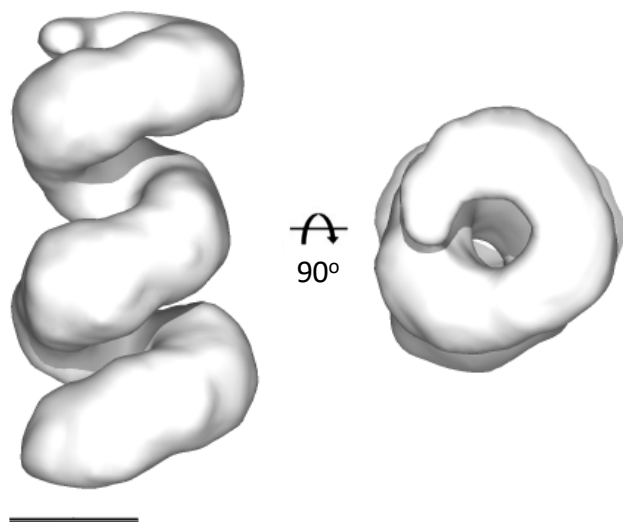

**B**

RNP asymmetric reconstruction = 14 Å

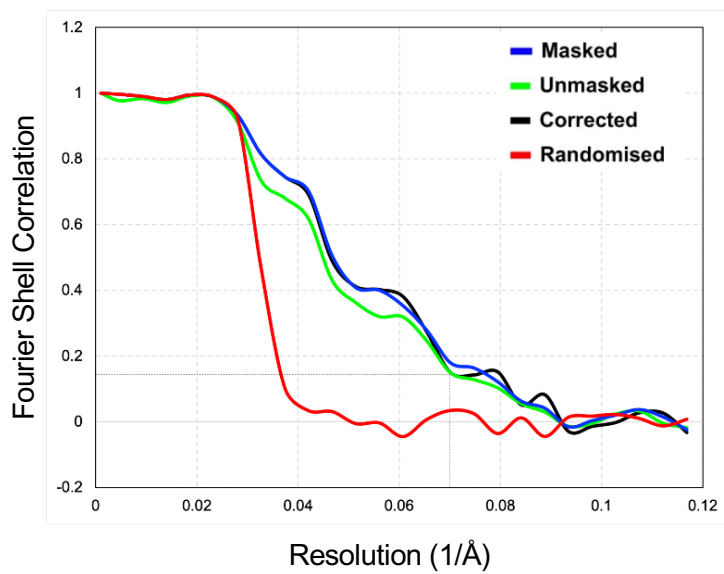

**C**

RNP symmetric reconstruction = 13 Å

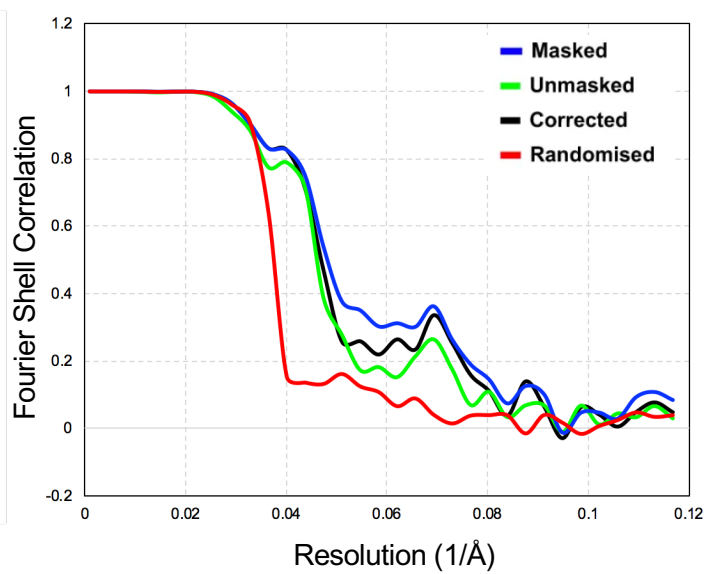

Supplement: FIG S4 [file mbio.01405-22-s0004.pdf]

**Figure S5**

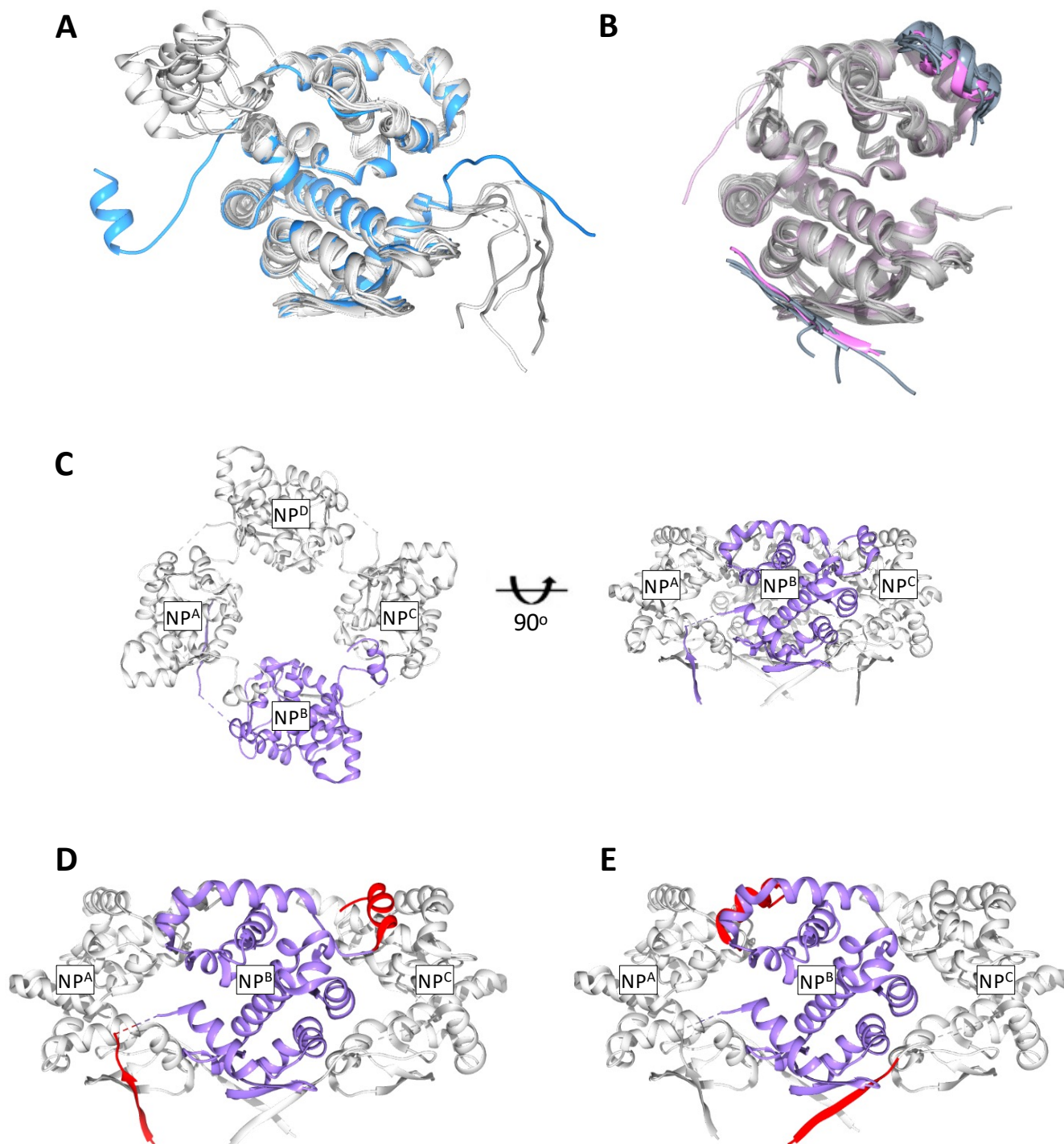

Supplement: FIG S5 [file mbio.01405-22-s0005.pdf]

**Figure S6**

**A**

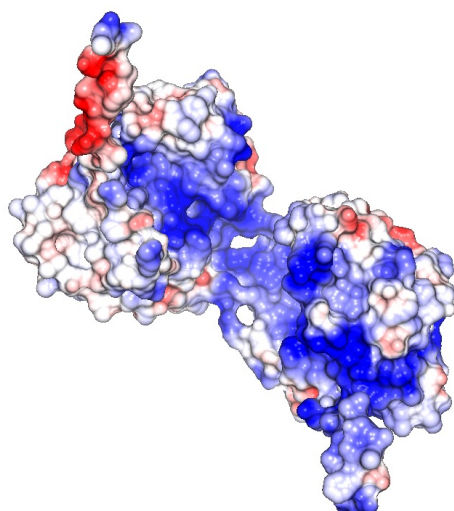

**B**

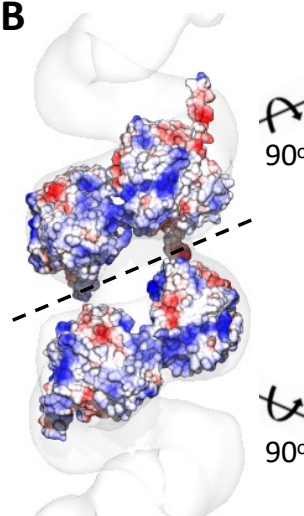

$90^\circ$

$90^\circ$

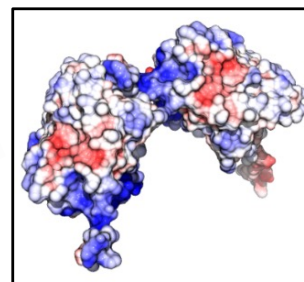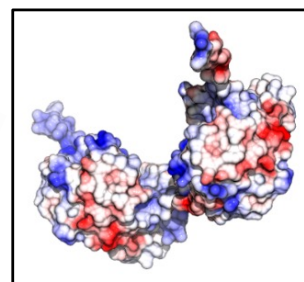

Supplement: FIG S6 [file mbio.01405-22-s0006.pdf]

**Figure S7**

**A**

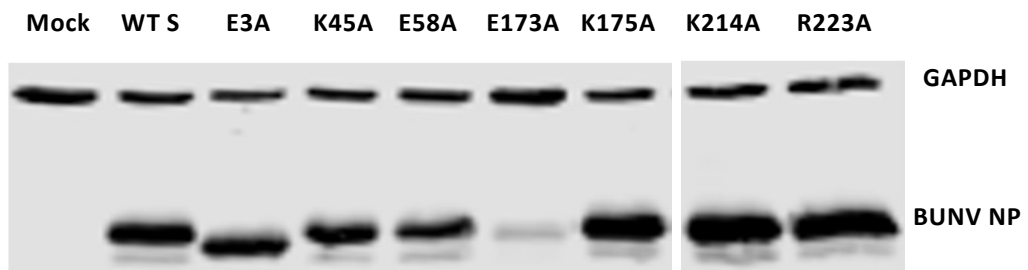

**B**

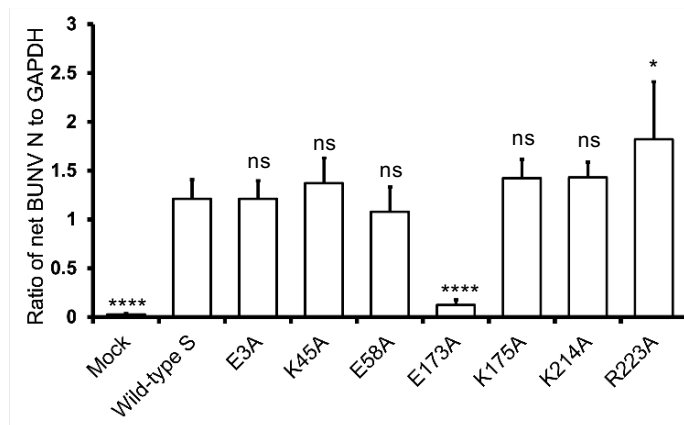

Supplement: FIG S7 [file mbio.01405-22-s0007.pdf]
