# Supplementary material for: The Native Orthobunyavirus Ribonucleoprotein Possesses a Helical Architecture
Source: mBio. 2022 Jun 28;13(4):e01405-22. doi: 10.1128/mbio.01405-22 (PMC9426602; doi:10.1128/mbio.01405-22)
Supplement: TABLE S1 [file mbio.01405-22-s0008.pdf]

Table S1

Helical model interactions

	##	Structure 1	Dist. [Å]	Structure 2
Hydrogen bonds	1	C:THR 62[N]	3.53	D:ILE 2[O]
	2	C:THR 62[OG1]	2.94	D:ILE 2[O]
	3	C:LEU 63[N]	3.76	D:GLU 3[OE2]
	4	C:ASN 64[N]	3.76	D:GLU 3[OE2]
	5	C:ASN 64[N]	3.16	D:LEU 4[O]
	6	C:LYS 45[NZ]	2.47	D:HIS 7[O]
	7	C:LYS 214[NZ]	2.73	D:GLU 173[OE2]
	8	C:ARG 223[NH2]	3.66	D:PRO 174[O]
	9	C:ARG 223[NH2]	3.73	D:GLU 178[OE2]
	10	C:GLU 58[OE2]	3.19	D:ILE 2[N]
	11	C:THR 62[O]	3.59	D:LEU 4[N]
	12	C:ASN 64[O]	3.14	D:PHE 6[N]
	13	C:ASN 217[O]	2.99	D:LYS 175[NZ]
Salt Bridges	1	C:LYS 214[NZ]	2.73	D:GLU 173[OE2]
	2	C:ARG 223[NH2]	3.73	D:GLU 178[OE2]
	3	C:GLU 58[OE1]	3.93	D:MET 1[N]
