# Supplementary material for: The Native Orthobunyavirus Ribonucleoprotein Possesses a Helical Architecture
Source: mBio. 2022 Jun 28;13(4):e01405-22. doi: 10.1128/mbio.01405-22 (PMC9426602; doi:10.1128/mbio.01405-22)
Supplement: TABLE S2 [file mbio.01405-22-s0009.pdf]

Table S2

3ZLA tetramer interactions

	##	Structure 1	Dist. [Å]	Structure 2
Hydrogen bonds	1	B:LEU 4[N]	3.31	A:THR 62[O]
	2	B:PHE 6[N]	2.95	A:ASN 64[O]
	3	B:GLN 168[NE2]	2.63	A:PHE 229[O]
	4	B:ILE 2[O]	3.16	A:THR 62[N]
	5	B:ILE 2[O]	3.19	A:THR 62[OG1]
	6	B:LEU 4[O]	3.05	A:ASN 64[N]
	7	B:PHE 6[O]	2.97	A:GLY 66[N]
	8	B:ASP 8[OD2]	3.39	A:ARG 40[NH1]
	9	B:GLU 178[OE2]	3.50	A:ASN 217[N]
Salt Bridges	1	B:ASP 8[OD2]	3.39	A:ARG 40[NH1]
